# Global roll-out of comprehensive policy measures may aid in bridging emissions gap

**DOI:** 10.1038/s41467-021-26595-z

**Published:** 2021-11-05

**Authors:** Heleen L. van Soest, Lara Aleluia Reis, Luiz Bernardo Baptista, Christoph Bertram, Jacques Després, Laurent Drouet, Michel den Elzen, Panagiotis Fragkos, Oliver Fricko, Shinichiro Fujimori, Neil Grant, Mathijs Harmsen, Gokul Iyer, Kimon Keramidas, Alexandre C. Köberle, Elmar Kriegler, Aman Malik, Shivika Mittal, Ken Oshiro, Keywan Riahi, Mark Roelfsema, Bas van Ruijven, Roberto Schaeffer, Diego Silva Herran, Massimo Tavoni, Gamze Unlu, Toon Vandyck, Detlef P. van Vuuren

**Affiliations:** 1 PBL Netherlands Environmental Assessment Agency, PO Box 30314, 2500 GH The Hague, the Netherlands; 2 Copernicus Institute of Sustainable Development, Utrecht University, Princetonlaan 8a, 3584 CB Utrecht, the Netherlands; 3 RFF‐CMCC European Institute on Economics and the Environment (EIEE), Centro Euro‐Mediterraneo sui Cambiamenti Climatici, Milan, 20144 Italy; 4 Centre for Energy and Environmental Economics (Cenergia), Energy Planning Programme (PPE), COPPE, Universidade Federal do Rio de Janeiro, Rio de Janeiro, Brazil; 5 Potsdam Institute for Climate Impact Research, Member of the Leibniz Association, P.O. Box 601203, 14412 Potsdam, Germany; 6 European Commission, Joint Research Centre (JRC), Seville, Spain; 7 Institute for Environmental Studies (IVM), Vrije Universiteit Amsterdam, Amsterdam, the Netherlands; 8 E3Modelling S.A., Panormou, 70-72 Athens, Greece; 9 International Institute for Applied Systems Analysis, Schlossplatz 1, A-2361 Laxenburg, Austria; 10 Department of Environmental Engineering, Kyoto University, C1-3 361 Kyotodaigaku Katsura, Nishikyoku, Kyoto city, Japan; 11 National Institute for Environmental Studies, 16-2 Onogawa, Tsukuba, Ibaraki, 305-8506 Japan; 12 Grantham Institute for Climate Change and the Environment, Imperial College London, Exhibition Road, London, SW72AZ United Kingdom; 13 Joint Global Change Research Institute, Pacific Northwest National Laboratory and University of Maryland, College Park, MD USA 20740; 14 Faculty of Economics and Social Sciences, University of Potsdam, August-Bebel-Str. 89, Potsdam, 14482 Germany; 15 Institute for Global Environmental Strategies, 2108-11 Kamiyamaguchi, Hayama, Kanagawa 240-0115 Japan; 16 Politecnico di Milano, Department of Management, Economics and Industrial Engineering, Milan, Italy

**Keywords:** Climate-change mitigation, Climate-change policy, Climate-change mitigation

## Abstract

Closing the emissions gap between Nationally Determined Contributions (NDCs) and the global emissions levels needed to achieve the Paris Agreement’s climate goals will require a comprehensive package of policy measures. National and sectoral policies can help fill the gap, but success stories in one country cannot be automatically replicated in other countries. They need to be adapted to the local context. Here, we develop a new Bridge scenario based on nationally relevant, short-term measures informed by interactions with country experts. These good practice policies are rolled out globally between now and 2030 and combined with carbon pricing thereafter. We implement this scenario with an ensemble of global integrated assessment models. We show that the Bridge scenario closes two-thirds of the emissions gap between NDC and 2 °C scenarios by 2030 and enables a pathway in line with the 2 °C goal when combined with the necessary long-term changes, i.e. more comprehensive pricing measures after 2030. The Bridge scenario leads to a scale-up of renewable energy (reaching 52%–88% of global electricity supply by 2050), electrification of end-uses, efficiency improvements in energy demand sectors, and enhanced afforestation and reforestation. Our analysis suggests that early action via good-practice policies is less costly than a delay in global climate cooperation.

## Abstract (web summary)

Comprehensive policy measures are needed to close the emissions gap between Nationally Determined Contributions and emissions goals of the Paris Agreement. Here the authors present a Bridge scenario that may aid in closing the emissions gap by 2030.

## Introduction

In the Paris Agreement, countries agreed to limit global warming to well below 2 °C, and preferably 1.5 °C^1^. For implementation, the Paris Agreement relies on mitigation action at the national level. These actions are communicated via nationally determined contributions (NDCs) and long-term strategies, containing each country’s pledged contribution to global mitigation. A key question is whether the collective action of all countries leads to the implementation of the Paris Agreement’s climate goals^2,3^. For this, countries agreed on a global stocktake process to periodically review collective progress and, if needed, stimulate additional efforts to meet the Paris Agreement’s global climate mitigation goals.

Several publications have already shown that the aggregated impact of NDCs is insufficient^4,5^. In addition, global emissions implied by nationally implemented policies are, collectively, even exceeding the global emissions levels projected under current NDCs^4^. This means that current NDCs and policies need to be strengthened. Scenarios from global integrated assessment models (IAMs) can provide guidance on how to do this. These include scenarios that provide information on how to implement reductions cost-optimally. However, in reality, it is not always possible to implement the measures included in these cost-optimal pathways^6^. For instance, influential societal actors might be able to block certain measures if they go against their interests. Market distortions can also make certain measures unattractive. Other solutions might lack societal support (e.g., carbon capture and storage), and also the rate at which a transition can be implemented may be slowed down (e.g., in the case of closing coal mines given the impact on coal miners and coal-dependent regions and communities). At the same time, however, there is also evidence of effective implementation of climate policies^7^. Here, good practice policies are defined as successfully implemented policies in one or more countries with a noticeable impact on greenhouse gas (GHG) emissions. In some cases, these policies are not even part of the cost-optimal mix suggested by models but could be easier to implement. It has been suggested that scaling up these good practice policies to other parts of the world might in the short-term be a more feasible and convincing strategy^8–13^.

First of all, history has shown that costs are only one factor influencing policy choices (see, e.g., Trutnevyte^14^, and the example of investments in renewable energy in the period that costs were still high). Other factors that influence policy choices include societal support, the influence of specific actors, and possible (perceived) co-benefits and trade-offs, including impacts on competitiveness. Second, such good practice policies have already been implemented in some countries, showing their effectiveness, at least in some places. Third, earlier work^15^ suggests that strengthening administrative and firm capabilities involved with monitoring, reporting and verification of emissions to support trading systems requires time and effort. Literature on policy sequencing^16,17^ shows how policies go through stages and at some point gain enough traction, experience, and political momentum to eventually move to efficient carbon pricing.

Fekete et al.^7^, Roelfsema et al.^8^, and Kriegler et al.^9^ investigated the impact of replicating such good practice policies in other parts of the world by focusing on global GHG emissions and indicators related to implementability (such as maximum annual average emissions reduction rate, carbon price increase per decade, or cumulative CCS deployment). Although helpful as a first step, this earlier work is limited by 1) the formulation of good practice policies at the global scale and 2) being based on a limited number of models. Better information on such good practice policies is needed to support the UNFCCC global stocktake in 2023.

Here, we build on the earlier work^7–9^, also going beyond relatively abstract cost-optimal pathways as guidance for policy-making by focusing on concrete policy measures that can be implemented to close the emissions gap. We do this for the first time using multiple models (both global and national) to assess a common set of reduction measures. These measures have been defined in consultation with national experts, making the scenarios more relevant (see Methods for details). The key scenario is referred to as the Bridge scenario, as it aims to bridge the gap between the ambition levels set out by countries by 2030 and those consistent with limiting global warming to 2 °C. This scenario includes a set of well-defined measures that can be implemented in the 2020–2030 period and go beyond the ambition of the NDCs (good practice policies), and that would still allow reaching the Paris climate goals by transitioning to a cost-optimal path towards 2 °C after 2030 (see Methods), assuming that governments prepare the ground for comprehensive (pricing) measures that are socially acceptable, e.g. through the use of revenues^18^. A focus on successfully implemented policies, as done in the Bridge scenario, will likely have near-term advantages in terms of political feasibility compared to an approach that focuses solely on cost-effectiveness (see above). The Bridge scenario, for example, allows to follow the steps identified in work on policy sequencing and thus move more smoothly than scenarios focusing on cost-effectiveness. The sequencing of policies can be attractive for other reasons as well. This allows, for instance, a gradual phase-in of climate policy per sector, e.g., to give households time to adjust. This concern applies particularly to investments related to residential energy use, where the lifetime of infrastructure typically extends beyond a few years. Additionally, the policy package that we apply is regionally differentiated, with higher-income countries taking more significant action in the 2020s. This can address some of the feasibility concerns observed in cost-optimal scenarios, allocating mitigation efforts to low-income countries in the near term (given the high potential for low-cost options, but with considerable feasibility concerns). We show that the Bridge scenario closes two-thirds of the emissions gap between NDC and 2 °C scenarios by 2030 and enables a pathway in line with the 2 °C goal when combined with more comprehensive pricing measures after 2030. Our analysis suggests that early action via these good-practice policies is less costly than a delay in global climate cooperation.

## Results

In order to discuss the possible impacts of the Bridge scenario, we compare it to four other scenarios, i.e., the impacts of current policies (CurPol), the conditional NDCs (NDCplus), and the models’ cost-optimal pathways towards 2 °C (starting immediately: 2Deg2020, and with a delay: 2Deg2030) (see Methods and Supplementary Information for more details). For the first two scenarios, the current policies and NDCs were extended beyond 2030 by assuming equivalent effort, i.e., by extrapolating the equivalent carbon price in 2030, using the GDP growth rate of the different regions up to 2050 for the extrapolation (see Supplementary Methods). For the Bridge scenario, the defined set of measures was implemented up to 2030 (Table 1) and a cost-optimal path towards 2 °C was implemented after 2030 (see Supplementary Methods). A full description of the scenarios and additional results can be found in the Supplementary Information. In the context of the global stocktake, here we focus on the results at the global level and several large countries, while more detailed national-level results by national models can be found elsewhere^11^.

**Table 1:** The good practice policies that were assumed to be replicated globally in the Bridge scenario, with differentiated targets for high-income and low-/medium-income countries, adapted from earlier analysis of good practice policies^7–9^.

Sector	Measure	High-income countries	Low-/medium-income countries	Other (differs per measure)
AFOLU (Agriculture, Forestry and Other Land Use)	Treat manure from livestock with anaerobic digesters—Reduction of CH_4_ emissions from manure, relative to 2015	33% by 2030	15% by 2030	
	Increase nitrogen use efficiency—Reduction of N_2_O emissions from fertilizer, relative to 2015	10% by 2030	5% by 2030	
	Selective breeding to reduce CH_4_ emissions from enteric fermentation—Emission factor reduction (CH_4_/tonne milk and/or beef) or emissions reduction, relative to 2015	10% by 2030	0% by 2030	
	Increase natural forest afforestation and reforestation—rates for three tiers (different than high- and low-income): % increase in forest area per year, for 2015–2030	Tier 1 (China, Latin America): 2%/year	Tier 2 (South & South East Asia, Sub-Saharan Africa, Australia): 1%/year	Tier 3 (Europe, Turkey, 23% of Russia, USA): 0.5% /year
	Halt natural forest deforestation	0 ha/year by 2030	0 ha/year by 2030	
Energy supply	No new installations of unabated coal power plants	By 2025	By 2030	
	Increase of the share of renewables in total electricity generation per year (starting in 2020, until 2050 and up to 50%, maximum)	1.4%-point increase per year	1.4%-point increase per year	
	Coal mine CH_4_ emissions recovery	30% by 2030	30% by 2030	
	Reduce venting and flaring of CH_4_ and CO_2_— emission reduction, relative to 2015	36% by 2030	36% by 2030	
Buildings	Improve final energy efficiency of appliances compared to 2015 (autonomous improvement as well as due to policy)	17% by 2030 (starting in 2018)	7% by 2030 (starting in 2025)	
	Improve final energy intensity of new residential and commercial buildings	22 & 30 kWh/(m^2^ yr) by 2025	22 & 30 kWh/(m^2^ yr) by 2035	EU: 35 & 40 kWh/(m^2^ yr) by 2025
	No new installations of oil boiler capacity in new and existing residential and commercial buildings	By 2030	By 2040	EU: by 2020
	Improve efficiency of existing buildings—Share of existing buildings being renovated	11% by 2030	6% by 2030	
Industry	Apply CCS—Carbon captured and stored as share of industry’s total CO_2_ emissions (model-dependent)	1.5% by 2030	1.5% by 2040	
	Improve final energy efficiency, relative to 2015	11% by 2030	6% by 2030	
	Reduce N_2_O emissions from adipic/acid production—reduction, relative to 2015	99% by 2030	99% by 2030	
Transport	Improve energy efficiency of aviation, starting in 2018	0.78% per year by 2030	0.78% per year by 2030	
	Improve average fuel efficiency of new passenger cars	38 km/l by 2030	27 km/l by 2030	
	Increase the share of non-fossil in new vehicle sales	50% by 2030	25% by 2030	China: 25% by 2025
Waste	Reduce CH_4_ emissions, relative to 2015	55% by 2030	28% by 2030	
Economy-wide	Carbon pricing—pathways for three tiers (different than high- and low-income)	Tier 1 (OECD, EU): 40 USD/tCO_2_ by 2030	Tier 2 (Russia, Eastern Europe, China, Korea, Latin America): 25 USD/tCO_2_ by 2030	Tier 3 (all others): 10 USD/tCO_2_ by 2030
	Reduce F-gas emissions, induced by policies, relative to 2015	60% by 2030	38% by 2030	

### A bridge over the emissions gap

**Fig. 1:**
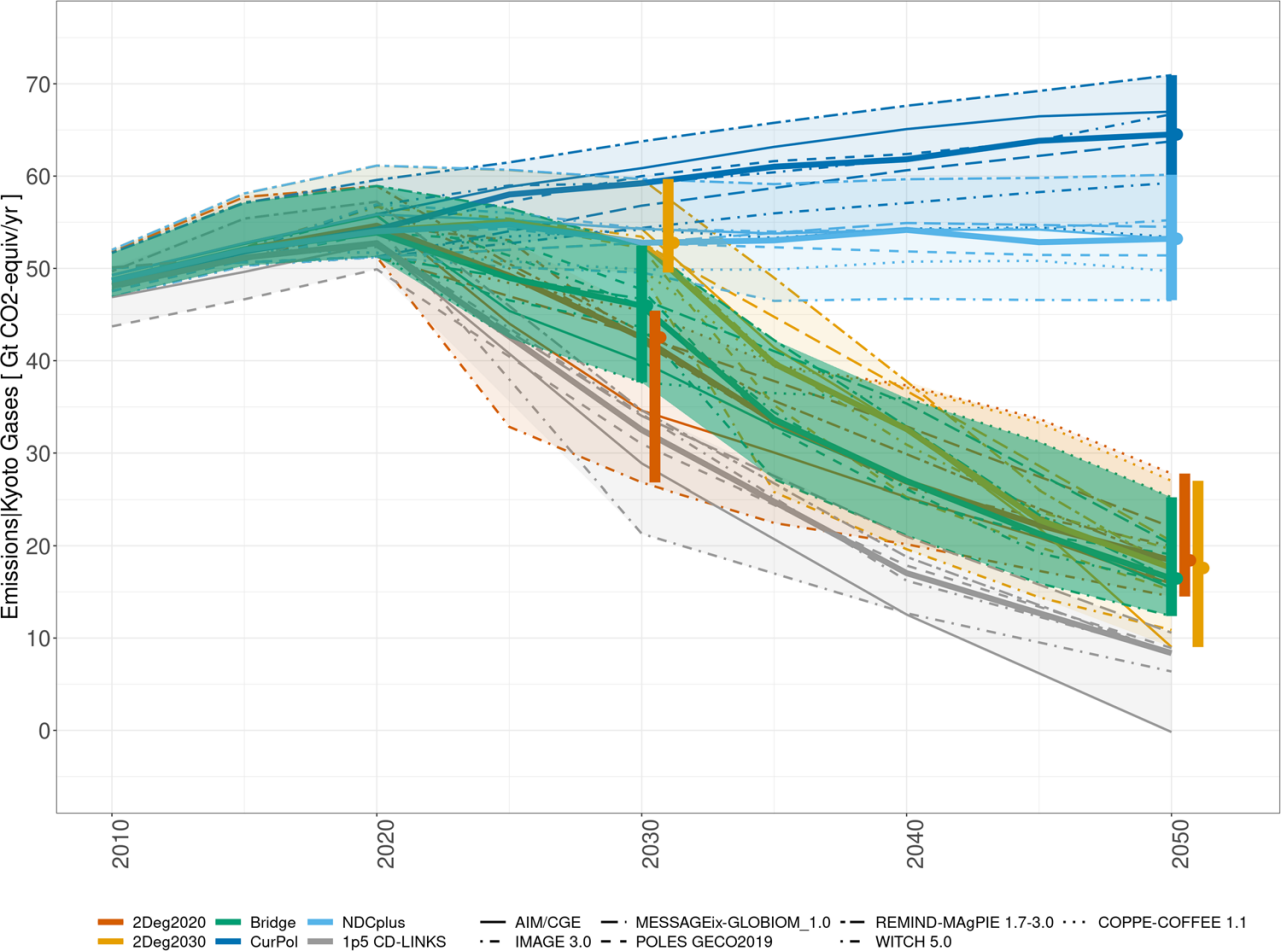
Global GHG emissions (Gt CO_2_eq/year) between 2010 and 2050, as projected by the global models. Vertical bars: model range in 2050. Circles: model median in 2050. Thick solid lines: median. Grey: 1.5 °C scenarios from the IPCC SR1.5 database are included for comparison (a selection was made to cover the same models as represented here, with most similar scenario set-up, i.e., the 1.5 °C scenarios developed in the CD-LINKS project^4^). Projections for the Bridge scenario without the carbon tax measure are shown in Supplementary Fig. 7, for NDCplus variant NDC_2050convergence in Supplementary Fig. 8, and for 2050—2100 in Supplementary Fig. 9.

The model outcomes (Fig. 1 and Supplementary Figs. 8 and 9) show that the CurPol and NDCplus scenarios both fall considerably short of the emission reductions needed to limit global warming to 2 °C (consistent with earlier work). In contrast, the good practice policies included in the Bridge scenario can reduce GHG emissions close to the needed levels in 2030, followed by a longer-term trajectory similar to the ambitious benchmark of 2Deg2020. The Bridge scenario has a less steep reduction than the 2Deg2030 scenario in the 2030s, offering a pathway that largely closes the 2030 emissions gap without adding substantial challenges in the 2030s and 2050s. The emissions gap is defined as the difference between the NDCplus scenario and the 2Deg2020 scenario (median: 11.8 GtCO_2_eq). The Bridge scenario closes that global emissions gap by 7.2 GtCO_2_eq or 60% (median, range 26–275%) by 2030 and compensates the slower start by a slightly deeper emission reduction in 2050, 106% (92–112%). Some recently submitted NDCs could not be considered as they came after the cut-off date of this work. Based on the Synthesis report by the UNFCCC^19^, global emissions levels under the NDCs would be 398 Mt CO_2_eq lower in 2030 when taking these into account (i.e., 3.4% of the median emissions gap found here and 5.5% of the 2030 emissions reductions under the Bridge scenario). Compared to a 1.5 °C scenario instead of 2Deg2020 (1.5 °C scenarios were not run here but included from the CD-LINKS project^4^ for comparison), the global emissions gap would be closed by 31% (21–57%) by 2030 and by 81% (71–85%) by 2050. The difference in 2030 emissions between the NDCplus and 2Deg2020 is closed by 16% in the USA, 49% in India, 56% in the EU and 68% in China.

Supplementary Fig. 1 shows the national rates of GHG emissions reductions in the Bridge scenario, compared to the CurPol, NDCplus, and cost-optimal cases (immediate: 2Deg2020 and delay: 2Deg2030). In contrast to the increase in GHG emissions under current policies in some countries, emissions decline everywhere in the Bridge scenario, especially in the 2030–2050 period. In most countries, the Bridge scenario shows smaller reductions than the immediate action 2Deg2020 scenario in the short term (2030), and smaller reductions than the 2Deg2030 scenario in the longer term (2050). As such, good practice policies can constitute an alternate pathway in line with limiting global warming to 2 °C, without relying on carbon pricing alone as in cost-optimal scenarios, while not significantly increasing the burden in the 2050s.

### Which measures have the largest effect on emissions?

**Fig. 2:**
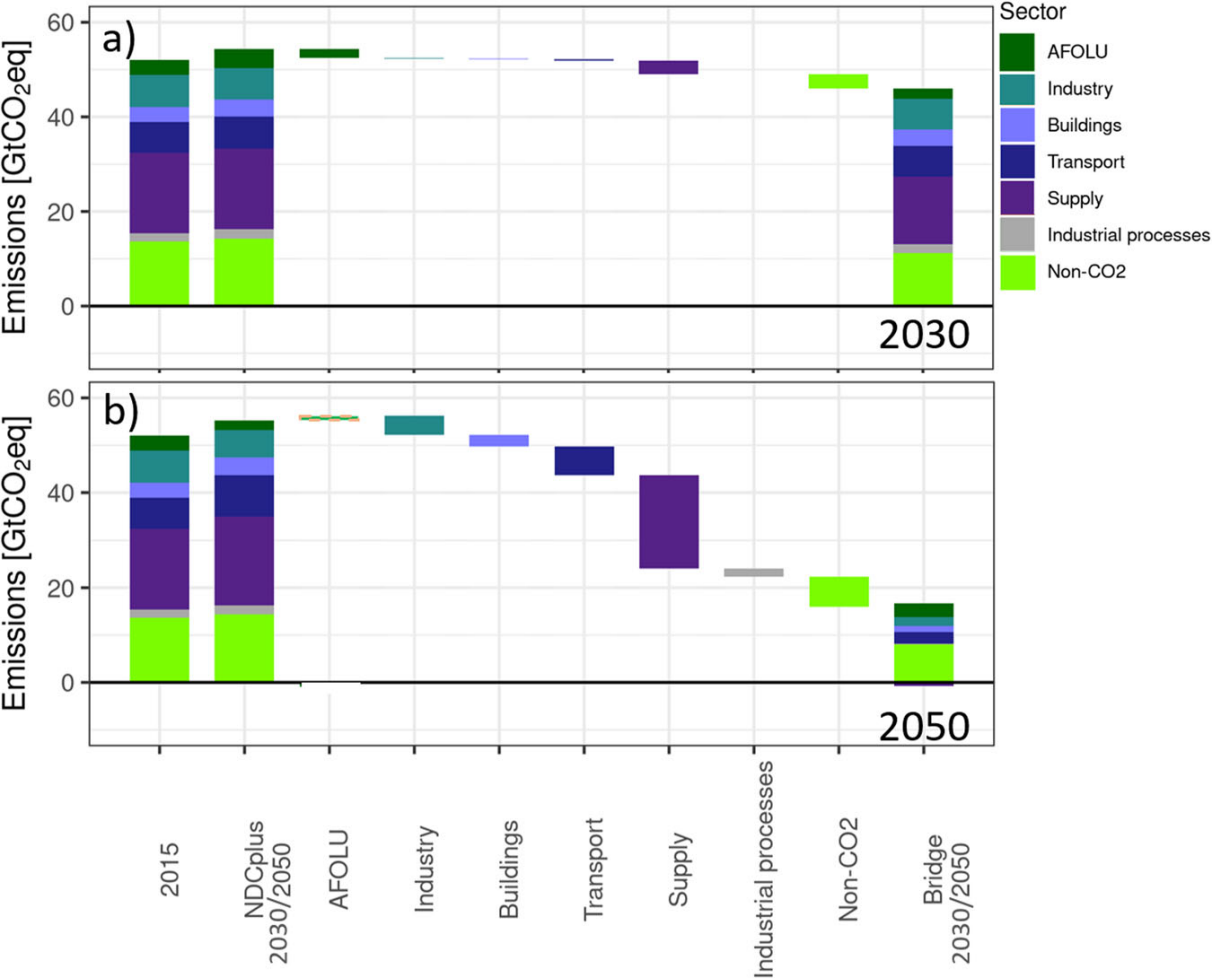
Contribution of each sector to emission reductions between the NDCplus and Bridge scenario (negative values denote an increase in emissions between NDCplus and Bridge, and are indicated with hashes). First bar: Emissions by sector in 2015. Second bar: emissions by sector in 2030 (panel a) and 2050 (panel b), under NDCplus. Third—ninth bar: emission reduction in AFOLU (Agriculture, Forestry, and Other Land Use), industry, buildings, transport, energy supply, industrial processes, non-CO_2_ emissions. Last bar: emissions by sector in 2030 (panel a) and 2050 (panel b), under Bridge. The IMAGE model is shown here as an illustrative example; full model ranges are shown in Table 2, while individual model results are shown in the SI (Supplementary Fig. 5). In addition, Supplementary Fig. 6 shows the sectoral contributions to emission reductions between the Bridge and 2Deg2020 scenarios in 2030.

The emissions gap between the NDCplus and 2Deg2020 scenarios amounts to approximately 12 GtCO_2_eq in 2030 (model median). The Bridge scenario closes this gap with 60% (a 7.2 GtCO_2_eq reduction). The energy supply sector (through higher renewable energy share, electrification, energy efficiency improvement) is the largest contributor to emissions reductions between the NDCplus and Bridge scenarios, both in 2030 and in 2050 (Fig. 2 and Table 2). In most models, mitigation of non-CO_2_ emissions, the transport sector (zero-carbon vehicles and efficiency improvements), and AFOLU (notably in 2030) also play an important role. This indicates potential to enhance ambition in specific areas, which will need to be explored at the national level.

**Table 2:** Share of sector in total GHG reduction from NDCplus to Bridge scenario (%), model range: minimum—maximum (median). AFOLU: Agriculture, Forestry, and Other Land Use.

Year	AFOLU	Industry	Buildings	Transport	Energy supply	Industrial Processes	Non-CO_2_
2030	−28.7–21.6 (7.8)	−10.1–14.8 (6.6)	−4.6–5.6 (2.3)	1.0–21.7 (8.9)	26.0–82.9 (50.1)	−0.2–5.4 (0.5)	2.9–50.6 (36.3)
2050	−2.4–11.8 (7.3)	7.9–31.4 (13.9)	2.9–9.5 (6.5)	6.5–15.6 (13.1)	34.6–49.8 (41.9)	0.1–8.1 (4.0)	9.6–20.0 (16.4)

### Changes in energy and land-use systems

**Fig. 3:**
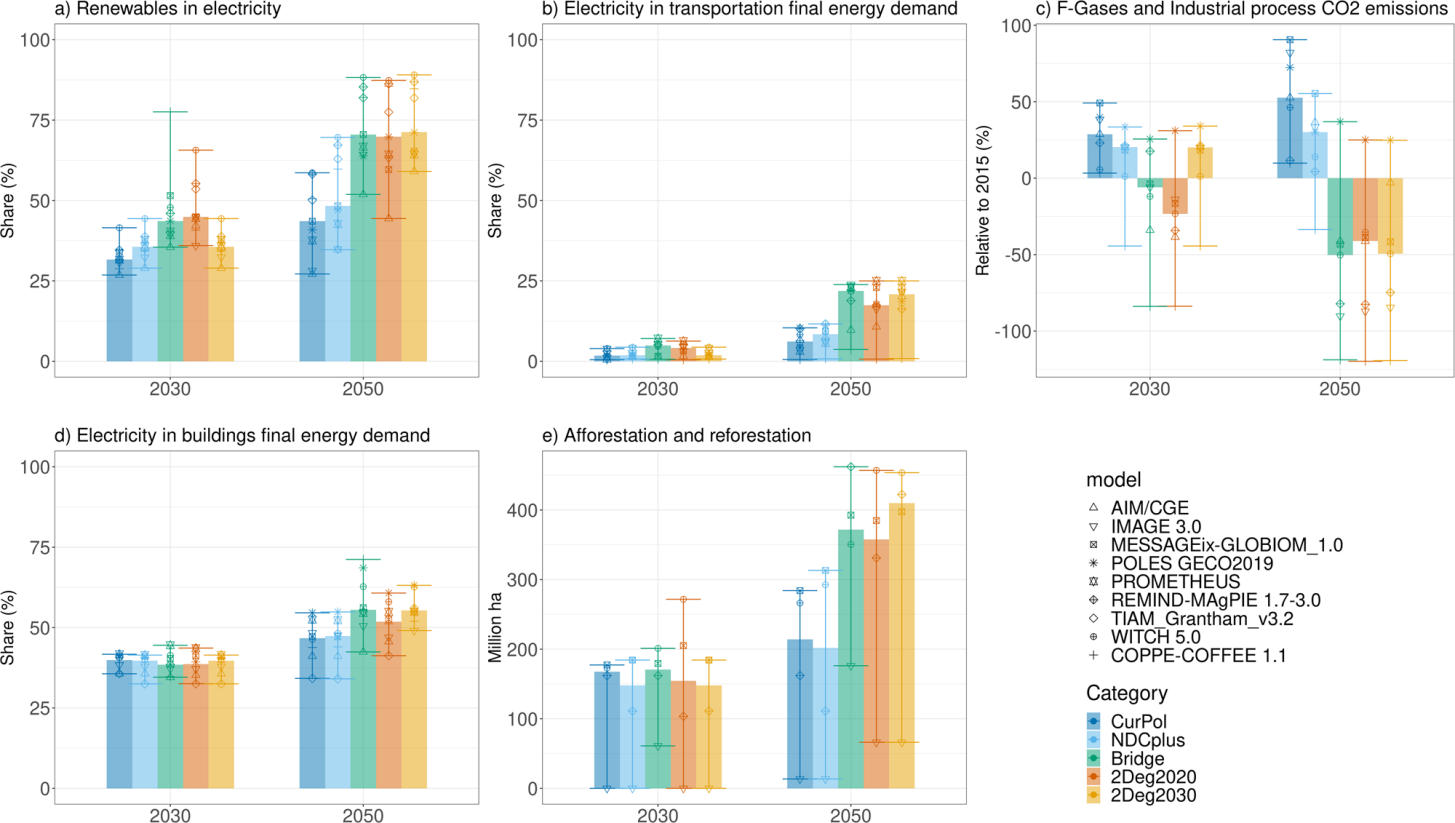
Projected changes in various indicators, for 2030 and 2050, for the CurPol, NDCplus, Bridge, 2Deg2020, and 2Deg2030 scenarios. Bars show model median, error bars show the full range, and symbols show individual model results. Panel **a** share of renewables in electricity production (%), panel **b** share of electricity in final energy demand of transportation (%), panel **c** Emissions of F-gases and industrial process CO_2_ emissions, relative to 2015 levels (%), panel **d** share of electricity in final energy demand of buildings (%), panel **e** total afforestation and reforestation (million ha).

Figure 3 shows projected changes in energy and land-use systems under five scenarios: CurPol, NDCplus, Bridge, 2Deg2020, and 2Deg2030. The Bridge scenario significantly increases mitigation action compared to the CurPol and NDCplus scenarios. In fact, on several indicators, the prescribed policies (Table 1 and Supplementary Tables) close the gap with the cost-optimal 2Deg2020 scenario almost completely. By 2050, the Bridge scenario is more ambitious than the 2Deg2020 scenario for many indicators, compensating for the delay with respect to the cost-optimal pathway. Figure 3 Panel a, for example, shows that the target to increase the renewable electricity share by 1.4% per year in the Bridge scenario leads to deployment far beyond the CurPol and NDCplus scenarios in 2050 (i.e., toward 70%, vs. around 50%), but similar to 2Deg2020 (in line with previous research^20^) and lower than 2Deg2030. In 2030, however, the Bridge scenario is similar to 2Deg2020, so it does not increase the global trend in terms of installing renewables in the short term (it may do so regionally, however, see Baptista et al^11^.). As a result of the assumed penetration of non-fossil fuelled vehicles, the Bridge scenario shows a significant increase in the share of electricity in transport, even more so in Bridge than in 2Deg2020 (Panel b). This starts in 2030, but manifests especially in 2050. However, in some models, the target to increase non-fossil fuelled vehicles actually leads to an increase of biofuel powered engines (Supplementary Fig. 2) rather than electrification (explaining the relatively large range), but less so than the 2Deg2030 scenario in 2050. Following CCS, efficiency improvement, and F-gas emission reduction targets in industry, industrial emissions (expressed as CO_2_ emissions from industrial processes as well as F-gases, panel c), are projected to decrease in Bridge slightly more so than in 2Deg2020 (by 2050). Because the measures in the buildings sector focus on energy efficiency improvements, the share of electricity in buildings (panel d) is not projected to change significantly in the 2030s, but Bridge makes up for that by 2050. Panel e shows that the afforestation policy leads to slightly more afforestation in 2030, followed by a large scale-up in 2050, but not as large as in 2Deg2030. As such, CO_2_ emissions from agriculture, forestry and other land-use (AFOLU) are projected to be reduced by 38% (model median) by 2030 and by 151% by 2050 in the Bridge scenario, relative to 2015 levels. Supplementary Fig. 3 shows the same indicators but for the NDCplus-convergence scenario instead of NDCplus: by 2050, the convergence scenario is closer to the Bridge scenario than NDCplus for most indicators. Supplementary Fig. 4, finally, shows the projected changes in the primary energy mix. Bridge sees lower total primary energy supply mainly due to the efficiency improvement and transport electrification measures, but not as low as 2Deg2020, and a shift from fossil fuels to renewable energy sources, especially by 2050. As a result of the scale-up of renewable energy, electrification of energy demand, and efficiency improvements, CO_2_ emissions from the energy sector are projected to decrease. The Bridge scenario has notable co-benefits: emissions of air pollutants such as black carbon, carbon monoxide, nitrogen oxides, organic carbon, sulphur, and volatile organic compounds are projected to decrease, compared to NDCplus (Supplementary Fig. 12).

### Costs of building the bridge

While the good practice policies may have benefits in terms of social and political acceptability, earlier work^9^ has highlighted that a set of regulatory measures may be more costly than a comprehensive carbon pricing scheme, leading to a noncost-optimal transition across regions and sectors. A uniform price signal would ensure that mitigation happens first where costs are lowest, leading to the overall most efficient outcome, in absence of other market failures. Although unlikely to be achieved globally, this stylised assumption therefore remains a useful benchmark. Furthermore, climate action as represented in the Bridge scenario implies a more gradual path for emission reductions in the period 2020–2030 compared to the immediate implementation of the cost-optimal policy (2Deg2020). This delay can further raise costs of the Bridge scenario, depending on the evolution of technology costs. The salience of a carbon price, however, may also raise opposition especially from low-income households facing energy poverty and food-insecurity^21^, carbon-intensive regions and vulnerable trade-exposed industries that may complicate or delay its implementation^22^. Arguably, the good practice policies included in the Bridge scenario face lower feasibility barriers and could speed up climate action compared to a scenario in which only cost-optimal policy measures are pursued. A fair evaluation of the costs of the Bridge scenario therefore involves two comparisons: one with the immediate and cost-optimal climate policy (2Deg2020), and one with a delayed implementation of uniform carbon pricing, starting in 2030 (2Deg2030) which requires more disruptive action to meet the 2 °C target.

**Fig. 4:**
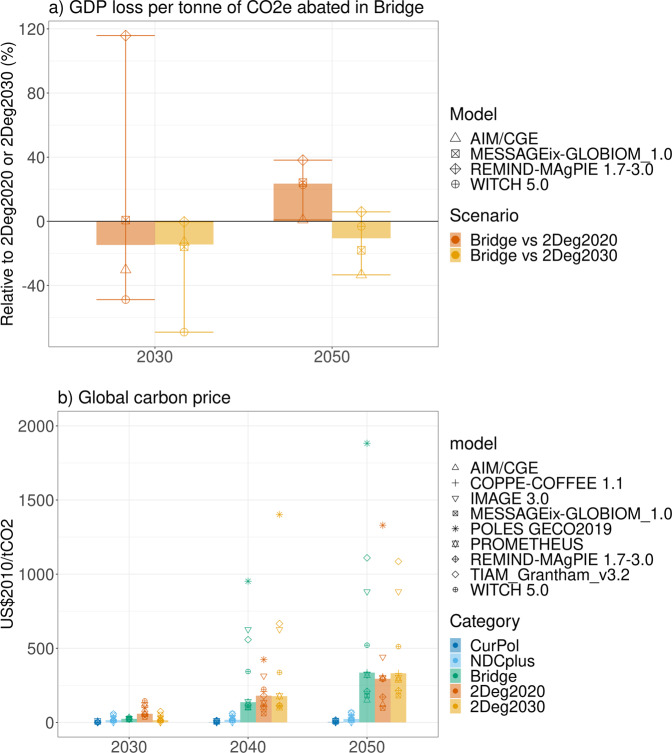
Cost indicators for the Bridge scenario, compared to the other scenarios. Panel **a** GDP (in market exchange rates, MER) loss (relative to the CurPol scenario) in Bridge, relative to 2Deg2020 (dark orange) and 2Deg2030 (yellow), for 2030 (left) and 2050 (right). Panel **b** Carbon price (US$2010/tCO_2_), in 2030, 2040 and 2050. Bars: median, error bars: full range, symbols: individual models.

Our results (Fig. 4) indicate that although the Bridge scenario raises policy costs (as expressed by GDP cost per tonne CO_2_eq abated relative to the Current Policies scenario) in 2050 by more than 20% (1–38%) compared to an immediate implementation of a cost-optimal 2 °C scenario with globally uniform carbon prices (2Deg2020), it has lower policy costs (Fig. 4a) and carbon prices (Fig. 4b and Supplementary Fig. 10) in the near term (2030). The Bridge scenario also outperforms a delayed 2 °C scenario (2Deg2030, see Supplementary Methods) with costs being more than 10% (-6–33%) lower in 2050. As such, our analysis suggests that early but noncost-optimal action is preferred over climate policy delay.

Interestingly, not all models in the ensemble agree on the size and sign of the trade-off between early and cost-optimal policy implementation. Multiple and counteracting effects are at play. Generally, good practice regulatory policies would raise costs particularly when the resulting energy system deviates strongly from the cost-optimal one. If the necessary changes are obvious, or when there are low-hanging fruits for climate policy, then a similar outcome may be achieved through regulation and carbon prices. The phase-out of coal and the scale-up of renewable power generation technologies^23–25^ may be an example that comes close (Supplementary Fig. 11 shows that investments in the electricity sector are projected to shift from fossil fuels to renewables). However, for other trade-offs, such as efficiency improvements versus fuel shift, or the allocation of emission reductions across sectors, a mix of regulatory measures that leads to an outcome resembling the cost-optimal one may be more difficult to achieve. Therefore, while regulatory policies can be a pragmatic entry-point for climate policy, cost-efficiency in the medium and long-term (beyond 2050) is more easily achieved via comprehensive carbon pricing schemes across all sectors and regions to avoid inter-sectoral and inter-regional leakage^12^. The costs of delaying climate action, on the other hand, depend on technological progress and the availability and scalability of negative emission technologies (NETs) in the future, among others^26^. For three out of four models that capture economic growth endogenously, the costs of delay outweigh the additional cost of regulatory good practice policies in 2050.

An advantage of the regulatory measures as implemented in the Bridge scenario is that carbon prices remain at lower levels in the near term, which may facilitate public acceptability and implementation of carbon pricing schemes with a broad sector coverage. If political consensus in favour of a comprehensive pricing scheme is not found over time, then a further intensification of the good practice policies may serve as a practical way forward to close the emissions gap. At the same time, the advantages of good practice policies in terms of acceptability may be challenged if ambitious climate targets bring cost elements to the forefront of the political debate.

Hence, our results suggest that a global roll-out of good practice policies can be a useful approach to close the emissions gap in the near term, while their role in climate policy in the longer term should be reconsidered in the context of a broader policy mix^16^, including carbon pricing^27^.

## Discussion

Parties to the Paris Agreement were supposed to submit updated NDCs and communicate their long-term strategies to the UNFCCC in 2020. Due to the COVID-19 pandemic, these timelines have been delayed and some countries have announced that they will not submit an updated NDC, while some others have not increased ambition in their updated NDC. However, scaling up climate ambition and action remains necessary to keep the Paris Agreement goals within reach. As the emissions gap seems hard to close, we built a set of relevant scenarios that may provide a pathway based on successful examples of policies. The mitigation measures were defined in a two-way interaction with country experts and assumptions were adjusted for different regions if necessary. These scenarios, especially the good practice policies (Bridge scenario), can support the ratcheting up of mitigation ambition of NDCs.

Although the granularity of the Bridge scenario has improved in terms of country differentiation compared to earlier studies, some limitations remain. In most cases, we only distinguished high-income and low-/middle-income countries, which (while an advance on existing scenarios) is only a second-best option. However, we did not find good arguments for country-specific groupings in policy categories other than afforestation, where the groupings are motivated by explicit afforestation targets in the respective NDCs. Differentiating by income group is a pragmatic approach that was approved by stakeholders from various countries. While the measures were assessed to be implementable, this might not always be the case when moving to the country-level. Therefore, Baptista et al^11^. discuss the same set of scenarios in the context of national feasibility considerations. Future work could further analyse the sustainable development implications of the Bridge scenario, for example, whether it has synergies with the goal to eradicate poverty. The other way around, a bridge scenario could be developed that takes the sustainable development goals as a starting point to identify nationally relevant areas for increased ambition in the 2030s.

Models implemented the set of measures in different ways. For example, not all models were able to implement all measures related to non-CO_2_, given their scope; while others show relatively cheap abatement and high potential to implement measures in the 2030s, resulting in a large range for the sector’s share in emission reductions. The ranges, however, do tell a robust story about the Bridge scenario in relation to the reference scenarios. Although set at a relatively low level, the carbon price measure was the single most effective policy in the 2030s. Removing it from the set of measures resulted in significantly higher emissions (Supplementary Fig. 7). However, as many countries or regions already have a form of carbon pricing, it deserves a spot in the selection of good practice policies, especially given the differentiated timelines and pricing levels assumed in the Bridge scenario. Finally, we have not considered the impact of the COVID-19 pandemic quantitatively, effectively assuming a full recovery without significant effect on long-term, global emissions^25^. The policy measures explored here, however, can inform governments that aim for green recovery packages^28^, by showing potential for ratcheting up mitigation ambition with a concrete set of measures.

We have shown that good practice policies can help to reach the 2 °C target in the long-term. They ensure closing the global emissions gap between NDCs and a cost-optimal 2 °C scenario by two-thirds (model median) by 2030. After 2030, more ambitious measures are needed. Such a Bridge scenario leads to lower energy sector emissions due to scale-up of renewable energy, electrification of energy demand, and efficiency improvements, and to lower land-use emissions due to afforestation—at levels and rates of change that are somewhat less than the 2Deg2020 case and less than the 2Deg2030 case. The scenario is still in a position that allows meeting the 2 °C goal, and, importantly, is less disruptive than 2Deg2030. However, although we included a wide set of good practice policies, they are jointly insufficient to put the world on track to meet the 1.5 °C target. The Bridge scenario further illustrates that good practice policies alone—without implementation of additional instruments such as a comprehensive carbon pricing scheme—are not enough to reach the 2 °C target. The Bridge scenario raises policy costs (as expressed by GDP loss per tonne of CO_2_ abated relative to the CurPol scenario) in 2050 by approximately 20% compared to a cost-optimal 2 °C scenario (2Deg2020). When put in perspective of economic growth in the coming three decades, this 20% cost increase implies that annual economic growth rates in the Bridge would be around 0.02 percentage points below the annual GDP growth in 2Deg2020. The Bridge scenario outperforms the delayed 2 °C scenario (2Deg2030) with global economic impacts being more than 10% lower in 2050. As such, early but noncost-optimal action is preferred over climate policy delay. In the absence of immediate, all-encompassing and ambitious climate policy measures, therefore, a global roll-out and successful implementation of good practice policies can put the world on track to a 2 °C-compatible pathway without posing large additional challenges.

In short, acting stringently on 2 °C (2Deg2020) is needed, but, collectively, we are not on track (NDCplus). If we do not strengthen collective action until 2030, the best chance at limiting global warming may be 2Deg2030. However, if we manage to accelerate action until 2030 (Bridge), major disruption can be avoided, even if we do not fully reach 2Deg2020. These results illustrate that short-term (2030) implementation of practical regulation-based policies is preferable over delayed climate action. At the same time, the institutional set-up should aim to avoid inefficient policy lock-in, as more efficient instruments may gain political and societal support over time.

## Methods

### Models

Both national and global model teams followed the same scenario protocol for comparability. The global models included here are: AIM/CGE^29^, COPPE-COFFEE^30^, IMAGE^31^, MESSAGEix-GLOBIOM^32^, POLES^33^, PROMETHEUS^34^, REMIND-MAgPIE^35^, TIAM-Grantham^36^, WITCH-GLOBIOM 5.0^37^. National-level results are presented in Baptista et al^11^.

### Scenarios

In line with the global stocktake, the ratcheting up mechanism has been applied in constructing the scenario protocol (see Supplementary Methods for the full protocol text and Supplementary Tables for the detailed lists of good practice policies). This means that the scenarios build upon one another in terms of ambition and modelling assumptions. The Current policies scenario is the least ambitious and the 2 °C scenario is the most ambitious.

#### Reference scenarios

The Current policies (CurPol) scenario incorporates middle of the road socioeconomic conditions throughout the century, based on the second marker baseline scenario from the Shared Socioeconomic Pathways (SSP2)^38^. It also assumes that climate, energy and land-use policies that are currently ratified are implemented (cut-off date 1 July 2019).

The NDC-plus scenario builds further upon the CurPol scenario and assumes that the conditional NDCs (both unconditional and conditional NDC actions) as submitted by April 2020 are implemented by 2030. After 2030, the scenario reflects continuation of effort (see below).

#### Bridge scenario

The Bridge scenario builds upon the CurPol scenario and assumes that certain good practice policies, which have shown to be effective in some countries^7–9^, will be implemented globally from 2020 until 2030 (Supplementary Table 1 in Supplementary Data lists the good practice policies, while Supplementary Table 2 gives an overview of their implementation in models, with the implemented shares ranging from 44% to 94%). After 2030, the Bridge scenario transitions to a 2 °C scenario following a cost-effective pathway (see below). The set of policies was defined in dialogue with national model teams, granting a more realistic scenario narrative (for more details, see the Supplementary Information). This was done in multiple rounds. First, national modelling teams responded to the proposed good practice policies (based on literature), considering whether these policies could be realistically implemented in their countries and, if not, what other target levels or years would be feasible. These teams cover Australia, Brazil, Canada, China, EU, India, Indonesia, Japan, Republic of Korea, Russia, United States; i.e., approximately 75% of global emissions. Second, the policy list was adjusted to differentiate country groups, regarding the timing and stringency of the targets. Third, some national models ran the refined scenarios and provided feedback, upon which the list was further refined. As such, we eventually defined two country groups (high-income and middle-/low-income), and in some cases three (adding Other, with different definition per measure), which were found to offer enough differentiation to be nationally relevant while still adhering to a common set of policy measures. Finally, all national and global model teams ran the agreed set of scenarios.

##### Country differentiation of good practice policies

A distinction is made between low/medium-income and high-income countries in terms of timing and stringency of good practice policy targets. The AFOLU sector’s measures are differentiated mostly in terms of stringency, not timing, considering the current differences in efficiency between high- and lower-income countries. Afforestation rates have a more specific country differentiation, based on NDC ambition. Energy supply measures are rather similar between countries as these are already more widespread, with the exception of coal phase-out, where low-income countries would need more time. Measures in the buildings sector are differentiated in terms of timing (overall energy intensity of buildings and oil boilers) as well as stringency (efficiency of appliances and renovation rate) given the different starting points and future service demand in country groups. For industry, the CCS measure was differentiated in timing only, as the development of the technology has a global nature, but its implementation may encounter different institutional barriers between higher and lower-income countries. For adipic/acid production, no differentiation was applied as significant emissions reductions are already technically possible. For F-gases, the differentiation is in line with the Kigali Agreement. Transport measures were not differentiated for aviation due to its global nature, but vehicle measures were assumed to be less stringent in low-income countries given different starting points. Given the more abundant use of landfilling in lower-income countries, reductions in methane emissions from waste were assumed to be smaller than in high-income countries.

##### Carbon pricing

Finally, as opposed to Fekete et al^7^., carbon pricing is included as good practice policy, although it may be considered as a top-down policy of different nature than the other policies. Carbon pricing and emission trading schemes have been successfully implemented in various countries. Furthermore, previous work^9^ highlights that good practice regulatory policies should be considered as complements to pricing-based approaches. In the simulations, the carbon price applies to all gases and sectors, hence represents an idealized view of carbon pricing schemes. It does not take the highest carbon price currently observed as starting point, but rather an approach in which countries were divided in three tiers with different carbon price levels and timelines to be most relevant to the countries represented here, and to better reflect the current status of pricing measures, such as ETS^39^. As a variant and to analyse the effect of this measure, some models ran an additional scenario excluding the carbon price measure (see Supplementary Fig. 7).

#### Post-2030 assumptions

The Bridge scenario follows the good practice policies until 2030, after which the scenario transitions smoothly to the 2 °C scenario by remaining within the carbon budget consistent with the 2 °C target (1000 GtCO_2_ for 2011–2100). This was implemented via a carbon price, with the scenario converging from the regionally differentiated 2030 carbon prices as prescribed to a global carbon price in 2050 that is in line with the 2 °C carbon budget. It is assumed that the gradual implementation of climate policy in the 2020–2030 period can build up enough momentum (and technology development) to move to a more comprehensive climate policy after 2030. The 2 °C (2Deg2020 and 2Deg2030) scenarios assume that an average temperature increase of 2 °C without overshooting is reached by 2100 in a cost-effective way (starting from 2020 in 2Deg2020 and from 2030 in 2Deg2030). National modelling teams used a carbon budget derived from the global carbon budget of 1000 Gt CO_2_ in the period 2011–2100 (including 2011 emissions), as done in CD-LINKS (https://www.cd-links.org/)^4^. This global carbon budget represents a 66% probability of keeping global warming below 2 °C. Carbon budgets have been revised since the CD-LINKS project in such a way that 1000 Gt is even more stringent than previously. Cumulative CO_2_ emissions in the 2 °C scenarios (2Deg2020, 2Deg2030, and Bridge) are not all exactly 1000 Gt, but range from 788 Gt CO_2_ to 1540 Gt CO_2_ (2011–2100), which is still within the range considered to be in line with 2 °C.

For the CurPol and NDC-plus scenarios, a continuation of efforts after the target year was assumed. This was implemented by extrapolating the equivalent carbon price in 2030, using the GDP growth rate of the different regions up to 2050. The equivalent carbon price represents the value of carbon that would yield the same emissions reduction as the NDC policies in a region. If a region has a carbon price of zero while implementing the NDC in 2030, a minimum carbon price of 1 $/tCO_2_ in 2030 was assumed. If a region has a negative carbon price in 2030, the trajectory resulting from 1 $/tCO_2_ was offset to the model’s 2030 starting point. For land use, a carbon price ceiling of $200/tCO_2_ was applied.

## Supplementary information

Supplementary Information File

## Data Availability

Model results can be found in the COMMIT scenario explorer: https://data.ece.iiasa.ac.at/commit/#/login?redirect=%2Fworkspaces. Policy relevant data is available in the Global Stocktake tool: https://themasites.pbl.nl/o/global-stocktake-indicators/#home. The scenario data generated in this study have been deposited in the Zenodo^40^ database under accession code 10.5281/zenodo.5163588. Source data are provided with this paper.

## Source data

Source Data

## Code availability

The models are documented on the common integrated assessment model documentation (https://www.iamcdocumentation.eu/index.php/IAMC_wiki), and several have published open source code (e.g. REMIND: https://github.com/remindmodel/remind), visualisation tools or detailed documentation (see references). The R-script that was used to generate the figures can be found on GitHub: https://github.com/Hel1vs/Bridge and Zenodo^41^: 10.5281/zenodo.5139955.

## References

[1] UNFCCC. *Paris Agreement: Decision 1/CP.17 - UNFCCC document FCCC/CP/2015/L.9/Rev.1*, http://unfccc.int/resource/docs/2015/cop21/eng/l09r01.pdf UNFCCC (2015).

[2] Rogelj J (2016). Paris Agreement climate proposals need a boost to keep warming well below 2 °C. Nature.

[3] Vrontisi Z (2018). Enhancing global climate policy ambition towards a 1.5 °C stabilization: a short-term multi-model assessment. Environ. Res. Lett..

[4] Roelfsema M (2020). Taking stock of national climate policies to evaluate implementation of the Paris Agreement. Nat. Commun..

[5] Fujimori S (2016). Implication of Paris Agreement in the context of long-term climate mitigation goals. SpringerPlus.

[6] Staub-Kaminski I, Zimmer A, Jakob M, Marschinski R (2014). Climate policy in practice: a typology of obstacles and implications for integrated assessment modeling. Clim. Change Econ..

[7] Fekete H (2021). A review of successful climate change mitigation policies in major emitting economies and the potential of global replication. Renew. Sustain. Energy Rev..

[8] Roelfsema M (2018). Reducing global GHG emissions by replicating successful sector examples: the ‘good practice policies’ scenario. Clim. Policy.

[9] Kriegler, E. et al. Short term policies to keep the door open for Paris climate goals. *Environ. Res. Lett*. **13**, 74022 (2018).

[10] Fekete, H. et al. *Impacts of good practice policies on regional and global greenhouse gas emissions*. (NewClimate Institute, PBL Netherlands Environmental Assessment Agency and International Institute for Applied Systems Analysis, Cologne, Germany; The Hague, the Netherlands; Laxenburg, Austria, 2015).

[11] Baptista, L. B. et al. *Good practice policies to bridge the emissions gap in key countries* (Submitted, 2021).

[12] Bertram C (2015). Complementing carbon prices with technology policies to keep climate targets within reach. Nat. Clim. Change.

[13] Höhne, N. et al. *Bridging the Gap: Enhancing Mitigation Ambition and Action at G20 Level and Globally. An Advance Chapter of The Emissions Gap Report 2019*. (United Nations Environment Programme, Nairobi, 2019).

[14] Trutnevyte E (2016). Does cost optimization approximate the real-world energy transition?. Energy.

[15] Zhang D (2019). Integrity of firms’ emissions reporting in China’s early carbon markets. Nat. Clim. Change.

[16] Pahle M (2018). Sequencing to ratchet up climate policy stringency. Nat. Clim. Change.

[17] Meckling J, Sterner T, Wagner G (2017). Policy sequencing toward decarbonization. Nat. Energy.

[18] Klenert D (2018). Making carbon pricing work for citizens. Nat. Clim. Change.

[19] UNFCCC Secretariat. *Nationally determined contributions under the Paris Agreement - Synthesis report by the secretariat.* (UNFCCC, Bonn, 2021).

[20] Luderer G (2018). Residual fossil CO2 emissions in 1.5–2 °C pathways. Nat. Clim. Change.

[21] Fujimori S (2019). A multi-model assessment of food security implications of climate change mitigation. Nat. Sustainability.

[22] Jenkins JD (2014). Political economy constraints on carbon pricing policies: What are the implications for economic efficiency, environmental efficacy, and climate policy design?. Energy Policy.

[23] IRENA. *Renewable power generation costs in 2019.* (International Renewable Energy Agency, Abu Dhabi, 2020).

[24] IEA. *Renewables 2020*. (IEA, Paris, 2020).

[25] IEA. *World Energy Outlook 2020*. (IEA, Paris, 2020).

[26] Daioglou, V. et al. Bioenergy technologies in long-run climate change mitigation: results from the EMF-33 study. *Clim. Change*, 10.1007/s10584-020-02799-y (2020).

[27] Oshiro, K. & Fujimori, S. Stranded investment associated with rapid energy system changes under the mid-century strategy in Japan. *Sustain. Sci.*10.1007/s11625-020-00862-2 (2020).

[28] Andrijevic M, Schleussner C-F, Gidden MJ, McCollum DL, Rogelj J (2020). COVID-19 recovery funds dwarf clean energy investment needs. Science.

[29] Fujimori, S. et al. AIM/CGE [basic] Manual. 1–87 (2012).

[30] COPPE/UFRJ. *Model Documentation - COFFEE-TEA*, https://www.iamcdocumentation.eu/index.php/Model_Documentation_-_COFFEE-TEA IAMC (2020).

[31] Stehfest, E., Van Vuuren, D. P., Bouwman, L. & Kram, T. *Integrated Assessment of Global Environmental Change with Model Description and Policy Applications IMAGE 3.0*. (PBL Netherlands Environmental Assessment Agency, 2014).

[32] Huppmann D (2019). The MESSAGEix Integrated Assessment Model and the ix modeling platform (ixmp): An open framework for integrated and cross-cutting analysis of energy, climate, the environment, and sustainable development. Environ. Model. Softw..

[33] Després, J., Keramidas, K., Schmitz, A., Kitous, A. & Schade, B. *POLES-JRC model documentation – 2018 update, EUR 29454 EN.* (Publications Office of the European Union, Luxembourg, 2018).

[34] Fragkos P, Kouvaritakis N (2018). Model-based analysis of Intended Nationally Determined Contributions and 2°C pathways for major economies. Energy.

[35] Aboumahboub, T. et al. *REMIND - REgional Model of INvestments and Development - Version 2.1.0*, https://www.pik-potsdam.de/research/transformation-pathways/models/remind PIK (2020).

[36] Loulou R, Labriet M (2008). ETSAP-TIAM: the TIMES integrated assessment model part I: model structure. Computational Manag. Sci..

[37] RFF-CMCC EIEE. *WITCH documentation*, https://doc.witchmodel.org/ (2019).

[38] Riahi K (2017). The Shared Socioeconomic Pathways and their energy, land use, and greenhouse gas emissions implications: An overview. Glob. Environ. Change.

[39] ICAP. *Emissions Trading Worldwide - Status Report 2021*. (International Carbon Action Partnership, Berlin, 2021).

[40] COMMIT consortium. COMMIT Scenario Explorer. COMMIT consortium (Zenodo). 10.5281/zenodo.5163588 (2021).

[41] van Soest, H. Global roll-out of comprehensive policy measures may aid in bridging emissions gap First release of the COMMIT Bridge repository. v. 1.0.0 (Zenodo, 2021 10.5281/zenodo.5139955).

