# Author Correction: Global roll-out of comprehensive policy measures may aid in bridging emissions gap

**DOI:** 10.1038/s41467-022-27969-7

**Published:** 2022-01-10

**Authors:** Heleen L. van Soest, Lara Aleluia Reis, Luiz Bernardo Baptista, Christoph Bertram, Jacques Després, Laurent Drouet, Michel den Elzen, Panagiotis Fragkos, Oliver Fricko, Shinichiro Fujimori, Neil Grant, Mathijs Harmsen, Gokul Iyer, Kimon Keramidas, Alexandre C. Köberle, Elmar Kriegler, Aman Malik, Shivika Mittal, Ken Oshiro, Keywan Riahi, Mark Roelfsema, Bas van Ruijven, Roberto Schaeffer, Diego Silva Herran, Massimo Tavoni, Gamze Unlu, Toon Vandyck, Detlef P. van Vuuren

**Affiliations:** 1 PBL Netherlands Environmental Assessment Agency, PO Box 30314, 2500 GH The Hague, the Netherlands; 2 Copernicus Institute of Sustainable Development, Utrecht University, Princetonlaan 8a, 3584 CB Utrecht, the Netherlands; 3 RFF‐CMCC European Institute on Economics and the Environment (EIEE), Centro Euro‐Mediterraneo sui Cambiamenti Climatici, Milan, 20144 Italy; 4 Centre for Energy and Environmental Economics (Cenergia), Energy Planning Programme (PPE), COPPE, Universidade Federal do Rio de Janeiro, Rio de Janeiro, Brazil; 5 Potsdam Institute for Climate Impact Research, Member of the Leibniz Association, P.O. Box 601203, 14412 Potsdam, Germany; 6 European Commission, Joint Research Centre (JRC), Seville, Spain; 7 Institute for Environmental Studies (IVM), Vrije Universiteit Amsterdam, Amsterdam, the Netherlands; 8 E3Modelling S.A., Panormou, 70-72 Athens, Greece; 9 International Institute for Applied Systems Analysis, Schlossplatz 1, A-2361 Laxenburg, Austria; 10 Department of Environmental Engineering, Kyoto University, C1-3 361 Kyotodaigaku Katsura, Nishikyoku, Kyoto city, Japan; 11 National Institute for Environmental Studies, 16-2 Onogawa, Tsukuba, Ibaraki, 305-8506 Japan; 12 Grantham Institute for Climate Change and the Environment, Imperial College London, Exhibition Road, London, SW72AZ United Kingdom; 13 Joint Global Change Research Institute, Pacific Northwest National Laboratory and University of Maryland, College Park, MD 20740 USA; 14 Faculty of Economics and Social Sciences, University of Potsdam, August-Bebel-Str. 89, Potsdam, 14482 Germany; 15 Institute for Global Environmental Strategies, 2108-11 Kamiyamaguchi, Hayama, Kanagawa 240-0115 Japan; 16 Politecnico di Milano, Department of Management, Economics and Industrial Engineering, Milan, Italy

**Keywords:** Climate-change mitigation, Climate-change policy, Climate-change mitigation

Correction to: *Nature Communications* 10.1038/s41467-021-26595-z, published online 5 November 2021.

The original version of this Article contained an error in Fig. 4b, in which a variable (IMAGE 3.0) for one scenario for the year 2050 was incorrectly reported/placed. This has been corrected in both the PDF and HTML versions of the Article.

