# Supplementary material for: Global roll-out of comprehensive policy measures may aid in bridging emissions gap
Source: Nat Commun. 2021 Nov 5;12:6419. doi: 10.1038/s41467-021-26595-z (PMC8571395; doi:10.1038/s41467-021-26595-z)
Supplement: Supplementary file 1 — Supplementary Information File [file 41467_2021_26595_MOESM1_ESM.pdf]

# Supplementary Information

# Global roll-out of comprehensive policy measures may aid in bridging emissions gap

Heleen L. van Soest[1,2]*, Lara Aleluia Reis[3], Luiz Bernardo Baptista[4], Christoph Bertram[5], Jacques Després[6], Laurent Drouet[3], Michel den Elzen[1,7], Panagiotis Fragkos[8], Oliver Fricko[9], Shinichiro Fujimori[10,11,9], Neil Grant[12], Mathijs Harmsen[1,2], Gokul Iyer[13], Kimon Keramidas[6], Alexandre C. Köberle[12], Elmar Kriegler[5], Aman Malik[5], Shivika Mittal[12], Ken Oshiro[10], Keywan Riahi[9], Mark Roelfsema[1,2], Bas van Ruijven[9], Roberto Schaeffer[4], Diego Silva Herran[11,14], Massimo Tavoni[3], Gamze Unlu[9], Toon Vandyck[6], Detlef P. van Vuuren[1,2]

[1] PBL Netherlands Environmental Assessment Agency, PO Box 30314, 2500 GH The Hague, the Netherlands

[2] Copernicus Institute of Sustainable Development, Utrecht University, Princetonlaan 8a, 3584 CB Utrecht, the Netherlands

[3]RFF-CMCC European Institute on Economics and the Environment, Milan 20144, Italy

[4] Centre for Energy and Environmental Economics (Cenergia), Energy Planning Programme (PPE), COPPE, Universidade Federal do Rio de Janeiro, Brazil

[5] Potsdam Institute for Climate Impact Research, Member of the Leibniz Association, P.O. Box 601203 , 14412, Potsdam, Germany

[6] European Commission, Joint Research Centre (JRC), Seville, Spain

[7] Institute for Environmental Studies (IVM), Vrije Universiteit Amsterdam, the Netherlands

[8] E3Modelling S.A., Panormou 70-72, Athens, Greece

[9] International Institute for Applied Systems Analysis, Schlossplatz 1, A-2361 Laxenburg, Austria

[10] Department of Environmental Engineering, Kyoto University, C1-3 361, Kyotodaigaku Katsura, Nishikyoku, Kyoto city, Japan

[11] National Institute for Environmental Studies, 16-2 Onogawa, Tsukuba, Ibaraki, 305-8506 Japan

[12] Grantham Institute, Imperial College London, Exhibition Road, London, SW7 2AZ, United Kingdom

[13] Joint Global Change Research Institute, Pacific Northwest National Laboratory and University of Maryland, College Park, MD, USA, 20740

[14] Institute for Global Environmental Strategies, 2108-11 Kamiyamaguchi, Hayama, Kanagawa, 240-0115 Japan

* Corresponding author:

Heleen L. van Soest (Heleen.vanSoest@pbl.nl)

# Supplementary Tables: implementation of good practice policies per model

The set of good practice policies was defined in dialogue with national model teams. In some cases, this process led to higher ambition than initially defined: for example, the target for the share of electric and hydrogen vehicles in new sales was brought forward by five years for China, and China was placed in the group of countries with highest assumed afforestation rates. In other cases, it led to a delay: for example, the target level for final energy intensity of buildings in the EU was adjusted, and for the USA, the carbon price was assumed to be introduced in 2025 rather than 2020.

*Supplementary Table 1: All good practice policies measures and their target values per country group. 2050 values only apply to the GPP scenario.*

See sheet ‘Supplementary Table 1’ in file “Supplementary Information – Supplementary Data.xlsx”.

*Supplementary Table 2: Implementation of the good practice policies listed in Table A 1, per model (percentages behind the model name indicate the share of the policies implemented, counting the orange and green cells, and excluding the proxy indicators)*

| ID | AIM (56%) | COFFEE (65%) | IMAGE (94%) | MESSAGEix_GLOBIOM (74%) | POLES (62%) | PROMETHEUS (53%) | REMIND-MAgPIE (71%) | WITCH (68%) | TIAM-Grantham_v3.2 (44%) |
|---|---|---|---|---|---|---|---|---|---|
| 1a | implemented | implemented | Included (non-$CO_2$ abatement factor) | agriculture policies not implemented | | agriculture policies not implemented | implemented (exact numbers as in Kriegler et al. 2018) | | Agricultural policies not implemented |
| 1b | implemented | implemented | Included (non-$CO_2$ abatement factor) | agriculture policies not implemented | | | implemented (exact numbers as in Kriegler et al. 2018) | | |
| 2a | implemented | not implemented | Included (non-$CO_2$ abatement factor) | agriculture policies not implemented | | | implemented (exact numbers as in Kriegler et al. 2018) | | |
| 2b | implemented | not implemented | Included (non-$CO_2$ abatement factor) | agriculture policies not implemented | | | implemented (exact numbers as in Kriegler et al. 2018) | | |
| 22 | | not implemented | Included (non-$CO_2$ abatement factor) | agriculture policies not implemented | | | | | |
| 24a | | Implemented | Included (non-$CO_2$ abatement factor) | agriculture policies not implemented | | | | | |

| ID | AIM (56%) | COFFEE (65%) | IMAGE (94%) | MESSAGEix_GLOBIOM (74%) | POLES (62%) | PROMETHEUS (53%) | REMIND-MAgPIE (71%) | WITCH (68%) | TIAM-Grantham_v3.2 (44%) |
|---|---|---|---|---|---|---|---|---|---|
| 24b | | not implemented | Included (non-$CO_2$ abatement factor) | agriculture policies not implemented | | | | | |
| 24c | | not implemented | Included (non-$CO_2$ abatement factor) | agriculture policies not implemented | | | | | |
| 3a | Adjusted the AEEI of builings sector to match protocol value. | Implemented | Included (Make appliance Unit Energy Consumption (kWh per appliance) improve according to the rate of the protocol) | implemented by using the proxy values | Implemented | proxy implementation finalised | implemented by using the proxy values | | Implemented |
| 3b | Adjusted the AEEI of builings sector to match protocol value. | Implemented | Included (Make appliance Unit Energy Consumption (kWh per appliance) improve according to the rate of the protocol) | implemented by using the proxy values | Implemented | proxy implementation finalised | implemented by using the proxy values | | Implemented |

| ID | AIM (56%) | COFFEE (65%) | IMAGE (94%) | MESSAGEix_GLOBIOM (74%) | POLES (62%) | PROMETHEUS (53%) | REMIND-MAgPIE (71%) | WITCH (68%) | TIAM-Grantham_v3.2 (44%) |
|---|---|---|---|---|---|---|---|---|---|
| 4a | | Implemented by using the proxy values | Included (through the previous measure + transitioned to fully-LED lighting + Reduce heating and cooling intensity (kJ/m2/HDD or CDD) according to the protocol (This is an exogenous variable in the version of TIMER which was used) + the next measure) | implemented by using the proxy values | Implemented | implemented | implemented by using the proxy values | | |

| ID | AIM (56%) | COFFEE (65%) | IMAGE (94%) | MESSAGEix_GLOBIOM (74%) | POLES (62%) | PROMETHEUS (53%) | REMIND-MAgPIE (71%) | WITCH (68%) | TIAM-Grantham_v3.2 (44%) |
|---|---|---|---|---|---|---|---|---|---|
| 4b | | Implemented by using the proxy values | Included (through the previous measure + transitioned to fully-LED lighting + Reduce heating and cooling intensity (kJ/m2/HDD or CDD) according to the protocol (This is an exogenous variable in the version of TIMER which was used) + the next measure) | implemented by using the proxy values | Implemented | implemented | implemented by using the proxy values | | |
| 4c | | - | - | implemented by using the proxy values | Implemented | implemented | | | |
| 4d | | - | - | implemented by using the proxy values | Implemented | implemented | | | |

| ID | AIM (56%) | COFFEE (65%) | IMAGE (94%) | MESSAGEix_GLOBIOM (74%) | POLES (62%) | PROMETHEUS (53%) | REMIND-MAgPIE (71%) | WITCH (68%) | TIAM-Grantham_v3.2 (44%) |
|---|---|---|---|---|---|---|---|---|---|
| 5a | | Implemented | Included (Deactivate coal and oil boilers for households. Only marginal capacity (existing capacity remains for their technical lifetime).) | implemented by using the proxy values | Implemented | Implemented | | | Implemented |
| 5b | | Implemented | Included (Deactivate coal and oil boilers for households. Only marginal capacity (existing capacity remains for their technical lifetime).) | implemented by using the proxy values | Implemented | Implemented | | | Implemented |

| ID | AIM (56%) | COFFEE (65%) | IMAGE (94%) | MESSAGEix_GLOBIOM (74%) | POLES (62%) | PROMETHEUS (53%) | REMIND-MAgPIE (71%) | WITCH (68%) | TIAM-Grantham_v3.2 (44%) |
|---|---|---|---|---|---|---|---|---|---|
| 6a | | Not implemented | Included (through the appliance energy consumption improvement + transitioned to fully-LED lighting + Reduce heating and cooling intensity (kJ/m2/HDD or CDD) according to the protocol (This is an exogenous variable in the version of TIMER which was used) + deactivation of coal and oil boilers for households) | implemented by using the proxy values | Implemented | Implemented | implemented by using the proxy values | | Not Implemented |

| ID | AIM (56%) | COFFEE (65%) | IMAGE (94%) | MESSAGEix_GLOBIOM (74%) | POLES (62%) | PROMETHEUS (53%) | REMIND-MAgPIE (71%) | WITCH (68%) | TIAM-Grantham_v3.2 (44%) |
|---|---|---|---|---|---|---|---|---|---|
| 6b | | Not implemented | Included (through the appliance energy consumption improvement + transitioned to fully-LED lighting + Reduce heating and cooling intensity (kJ/m2/HDD or CDD) according to the protocol (This is an exogenous variable in the version of TIMER which was used) + deactivation of coal and oil boilers for households) | implemented by using the proxy values | Implemented | Implemented | implemented by using the proxy values | | Not Implemented |
| 25 | | not implemented | - | implemented by using the proxy values | Implemented | implemented | | | Implemented |
| 7 | implemented | implemented | Included | implemented. | Implemented | implemented | implemented | | Implemented |

| ID | AIM (56%) | COFFEE (65%) | IMAGE (94%) | MESSAGEix_GLOBIOM (74%) | POLES (62%) | PROMETHEUS (53%) | REMIND-MAgPIE (71%) | WITCH (68%) | TIAM-Grantham_v3.2 (44%) |
|---|---|---|---|---|---|---|---|---|---|
| 8 | implemented | implemented | Included (as no coal-fired power plant; 420 $gCO_2/kWh$) | implemented | Implemented | implemented | implemented | | Implemented |
| 9a | Adjusted the preference for renewables | implemented | Included (premium factors and forced capacity shares) | implemented | Implemented | implemented | implemented | | Implemented |
| 9b | Adjusted the preference for renewables | implemented | Included (premium factors and forced capacity shares) | implemented | Implemented | implemented | implemented | | Implemented |
| 10a | implemented | not implemented | Included (F-gas specific tax) | implemented | | | implemented | | Not implemented (TIAM_Grantham not covering F-Gases in this analysis) |
| 10b | implemented | not implemented | Included (F-gas specific tax) | implemented | | | implemented | | Not implemented (TIAM_Grantham not covering F-Gases in this analysis) |
| 11 | implemented | implemented | Included (non-$CO_2$ abatement factor) | implemented | Implemented | | | | Not implemented |

| ID | AIM (56%) | COFFEE (65%) | IMAGE (94%) | MESSAGEix_GLOBIOM (74%) | POLES (62%) | PROMETHEUS (53%) | REMIND-MAgPIE (71%) | WITCH (68%) | TIAM-Grantham_v3.2 (44%) |
|---|---|---|---|---|---|---|---|---|---|
| 12a | implemented | implemented partially (% value not possible in all regions in 2030) | Included (non-$CO_2$ abatement factor) | not implemented due to model restriction | | | | | Not implemented |
| 12b | implemented | implemented | Included (non-$CO_2$ abatement factor) | not implemented due to model restriction | | | | | Not implemented |
| 26 | | implemented | Included (non-$CO_2$ abatement factor) | | | | | | Implemented |
| 13a | | implemented | - | implemented | Implemented | implemented | implemented (Approx. 200 $MtCO_2$ /year CCS in industry.) | | Implemented |
| 13b | | implemented | - | implemented | Implemented | | | | Implemented |
| 14a | Adjusted the AEEI of industry sector to match protocol value. | not implemented | Included (added efficiency factor) | implemented by using the proxies | | implemented | implemented by using the proxies | | Implemented |
| 14b | Adjusted the AEEI of industry sector to match | not implemented | Included (added efficiency factor) | | | implemented | implemented by using the proxies | | Implemented |

COMMIT

| ID | AIM (56%) | COFFEE (65%) | IMAGE (94%) | MESSAGEix_GLOBIOM (74%) | POLES (62%) | PROMETHEUS (53%) | REMIND-MAgPIE (71%) | WITCH (68%) | TIAM-Grantham_v3.2 (44%) |
|---|---|---|---|---|---|---|---|---|---|
| | protocol value. | | | | | | | | |
| 15 | implemented | not implemented | Included (non-$CO_2$ abatement factor) | implemented | Implemented | | | | Not implemented |
| 16 | | implemented | Included (approximation, by reduced addtinal deforestation) | not implemented | | | implemented (10 million ha/year afforestation) | | Not implemented |
| 17 | | implemented | Included (approximation, by reduced addtinal deforestation) | not implemented | | | implemented (End natural forest loss;) | | Not implemented |
| 27 | | not implemented | - | not implemented | | | | | Not implemented |
| 18a | | implemented | Included (added efficiency factor) | implemented by using proxies | Implemented | implemented | implemented by using proxies | | Implemented |
| 18b | | implemented | Included (added efficiency factor) | implemented by using proxies | Implemented | implemented | implemented by using proxies | | Implemented |

| ID | AIM (56%) | COFFEE (65%) | IMAGE (94%) | MESSAGEix_GLOBIOM (74%) | POLES (62%) | PROMETHEUS (53%) | REMIND-MAgPIE (71%) | WITCH (68%) | TIAM-Grantham_v3.2 (44%) |
|---|---|---|---|---|---|---|---|---|---|
| 19a | | implemented | Included (added efficiency factor) | implemented by using proxies | Implemented | Implemented by using proxies | implemented by using proxies | | Implemented |
| 19b | | - | - | implemented by using proxies | Implemented | implemented | implemented by using proxies | | Implemented |
| 19c | Adjusted AEEI in passenger transport to match final energy proxy values from IMAGE. | - | - | implemented by using proxies | Implemented | implemented | implemented by using proxies | | Implemented |
| 20 | | implemented. But we do also consider the sales of ethanol fuel cell vehicles | Included (by premium factors on vehicles) | implemented by using proxies | Implemented | implemented | implemented | | Implemented |
| 21a | implemented | not implemented | Included (non-$CO_2$ abatement factor) | implemented | Implemented | | | | Not implemented |
| 21b | implemented | not implemented | Included (non-$CO_2$ abatement factor) | implemented | Implemented | | | | Not implemented |

# Supplementary Figures

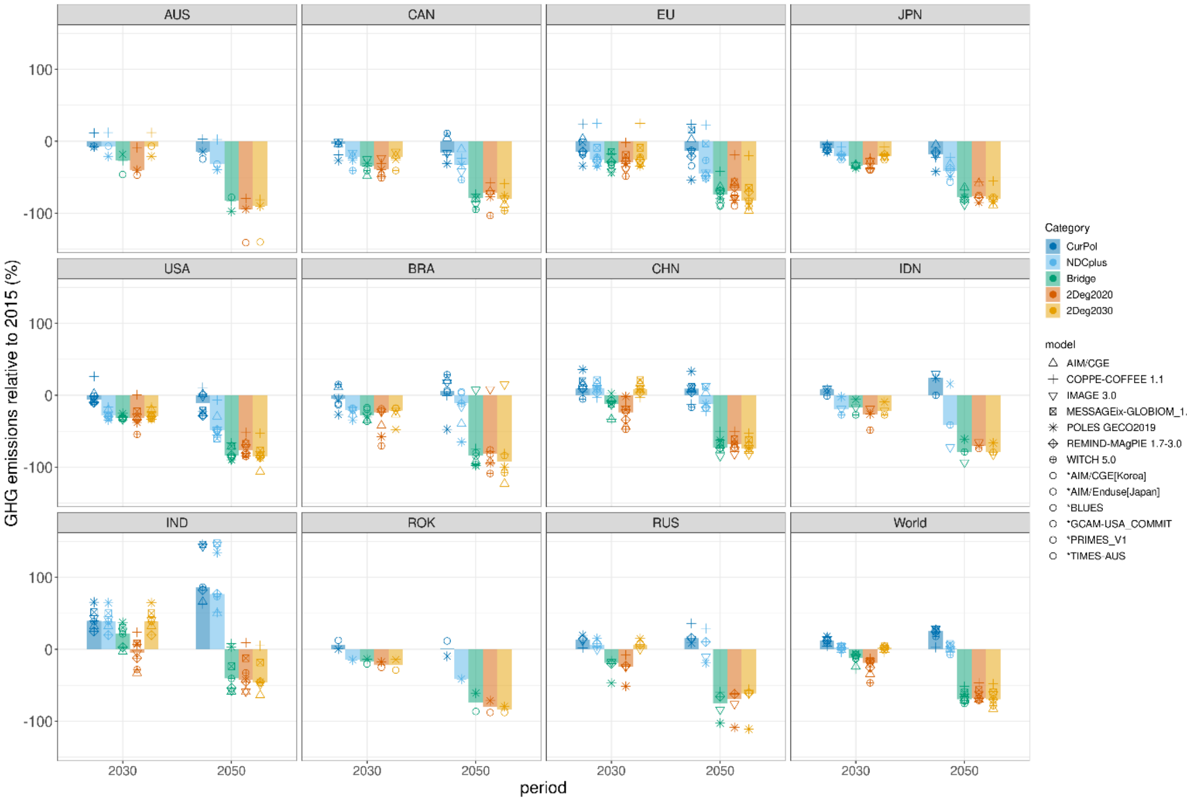


***Supplementary Figure 1: GHG emissions, relative to 2015 (%),*** *per region (panel), for 2030 and 2050 (X-axis), for the CurPol, NDCplus, Bridge, 2Deg2020, and 2Deg2030 scenarios (colours). Bars indicate model median, symbols the individual model results. AUS: Australia, CAN: Canada, EU: European Union, JPN: Japan, USA: United States of America, BRA: Brazil, CHN: China, IDN: Indonesia, IND: India, ROK: Republic of Korea (South Korea), RUS: Russian Federation.*

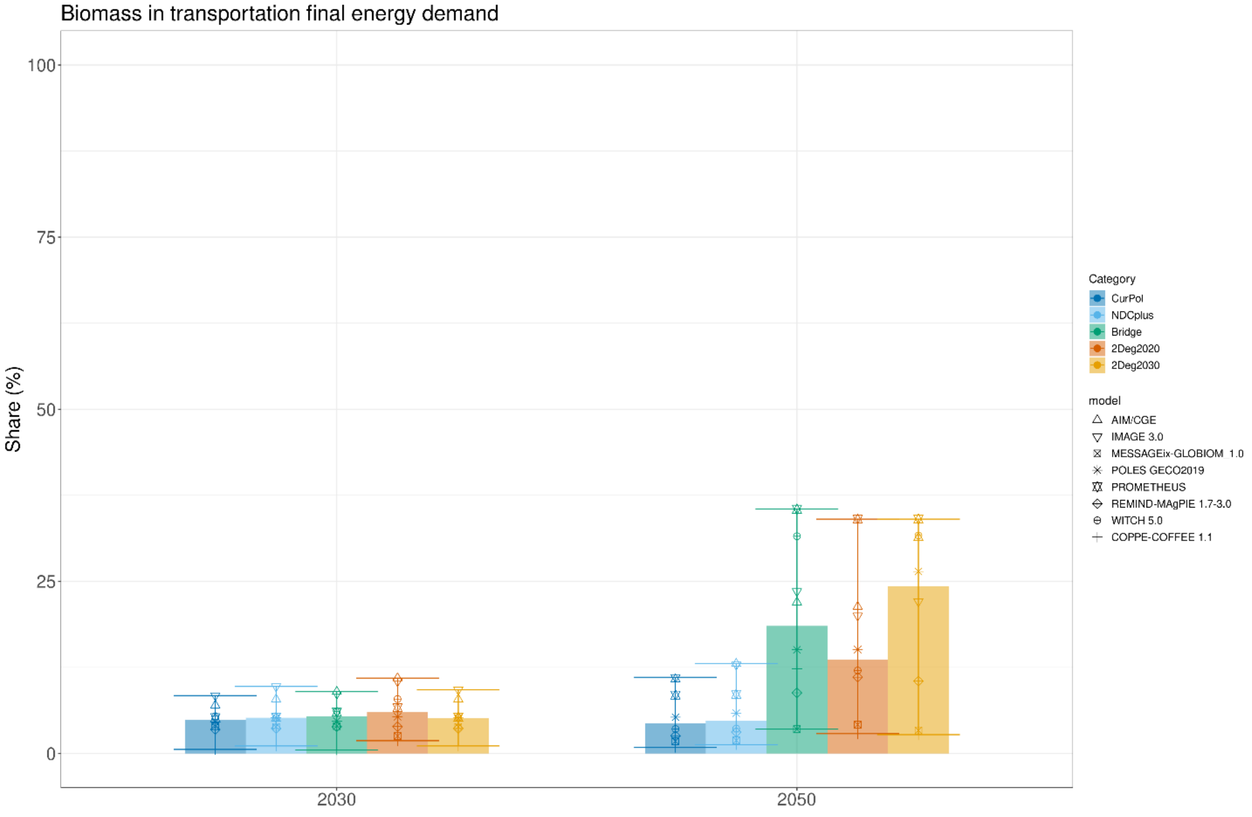


*Supplementary Figure 2: Projected changes in the share of liquid biomass in final energy demand of transportation (%), for 2030 and 2050, for the CurPol, NDCplus, Bridge, 2Deg2020, and 2Deg2030 scenarios. Bars show model median, error bars show the full range, and symbols show individual model results.*

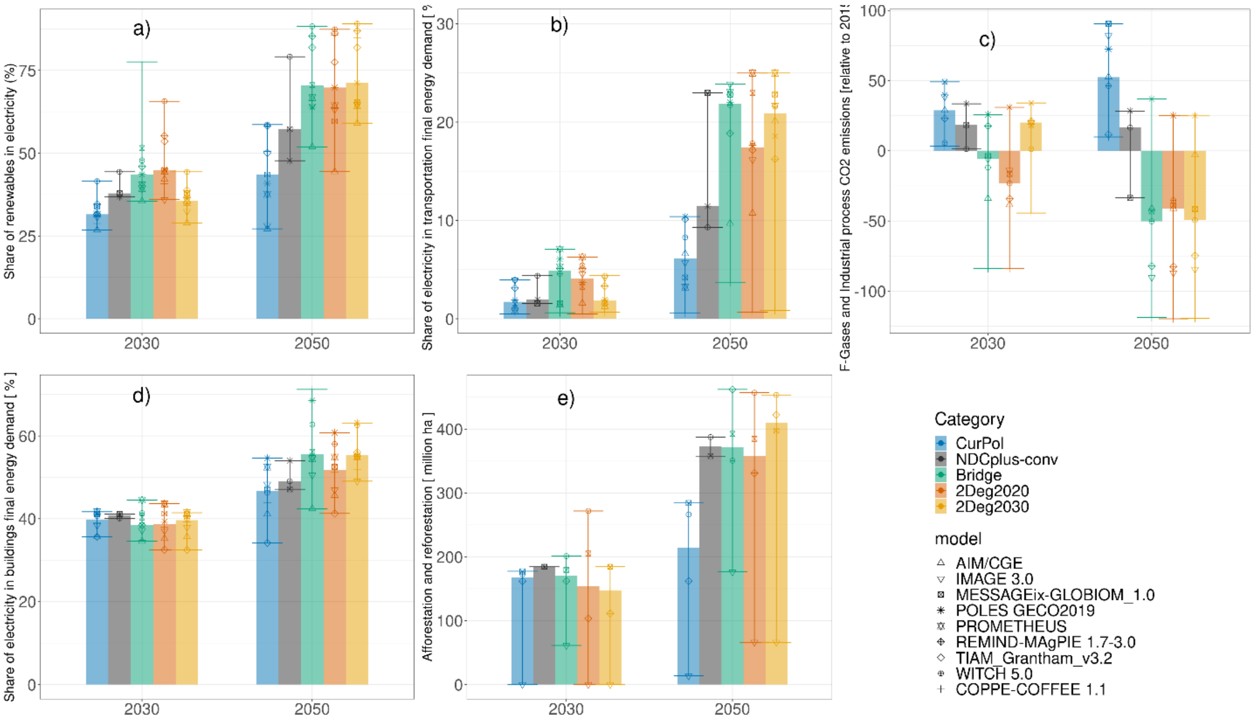


*Supplementary Figure 3: Projected changes in various indicators, for 2030 and 2050,* *for the CurPol, NDCplus-convergence, Bridge, 2Deg2020, and 2Deg2030 scenarios. Bars show model median, error bars show the full range, and symbols show individual model results. Panel a) share of renewables in electricity production (%), panel b) share of electricity in final energy demand of transportation (%), panel c) Emissions of F-gases and industrial process $CO_2$ emissions, relative to 2015 levels (%), panel d) share of electricity in final energy demand of buildings (%), panel e) total afforestation and reforestation (million ha).*

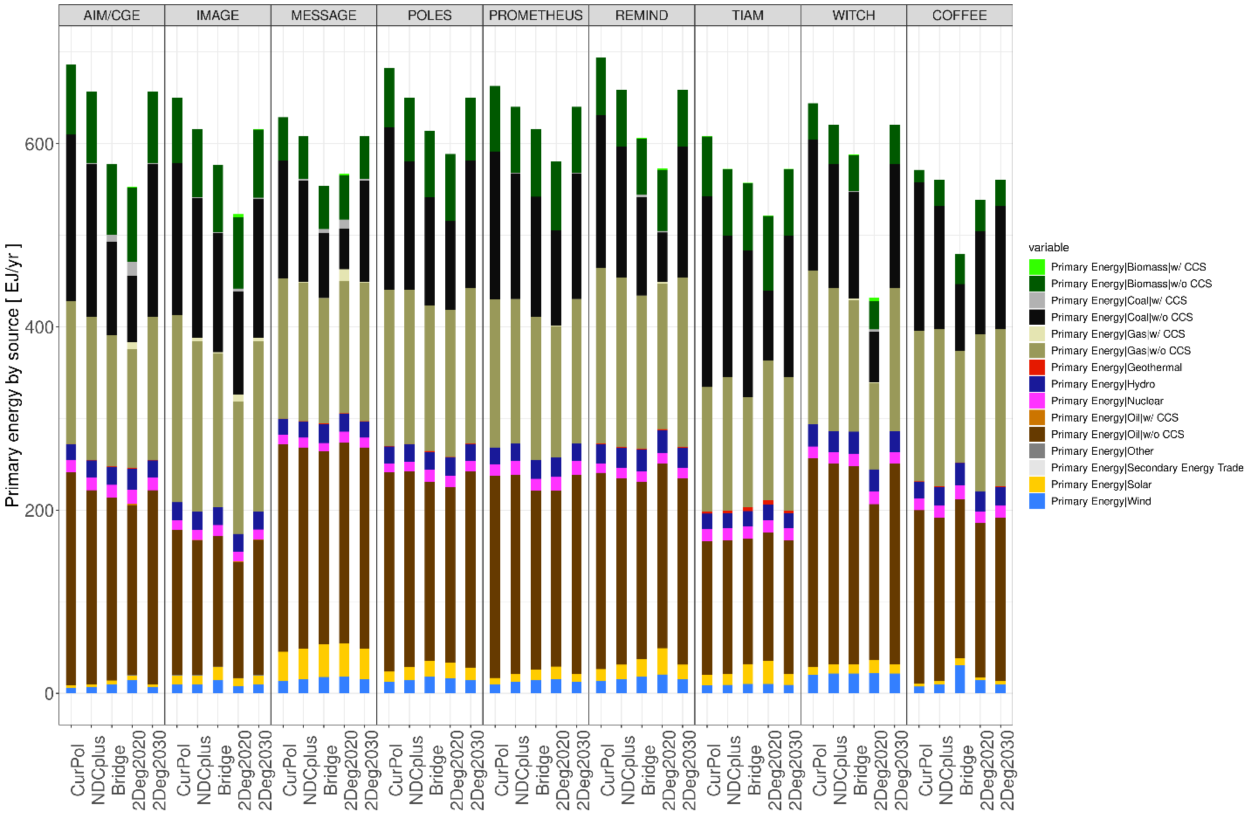


*Supplementary Figure 4: Primary energy production in 2030 by source*, *per model, for the CurPol, NDCplus, Bridge, 2Deg2020, and 2Deg2030 scenarios. w/: with, w/o: without. CCS: Carbon Capture and Storage.*

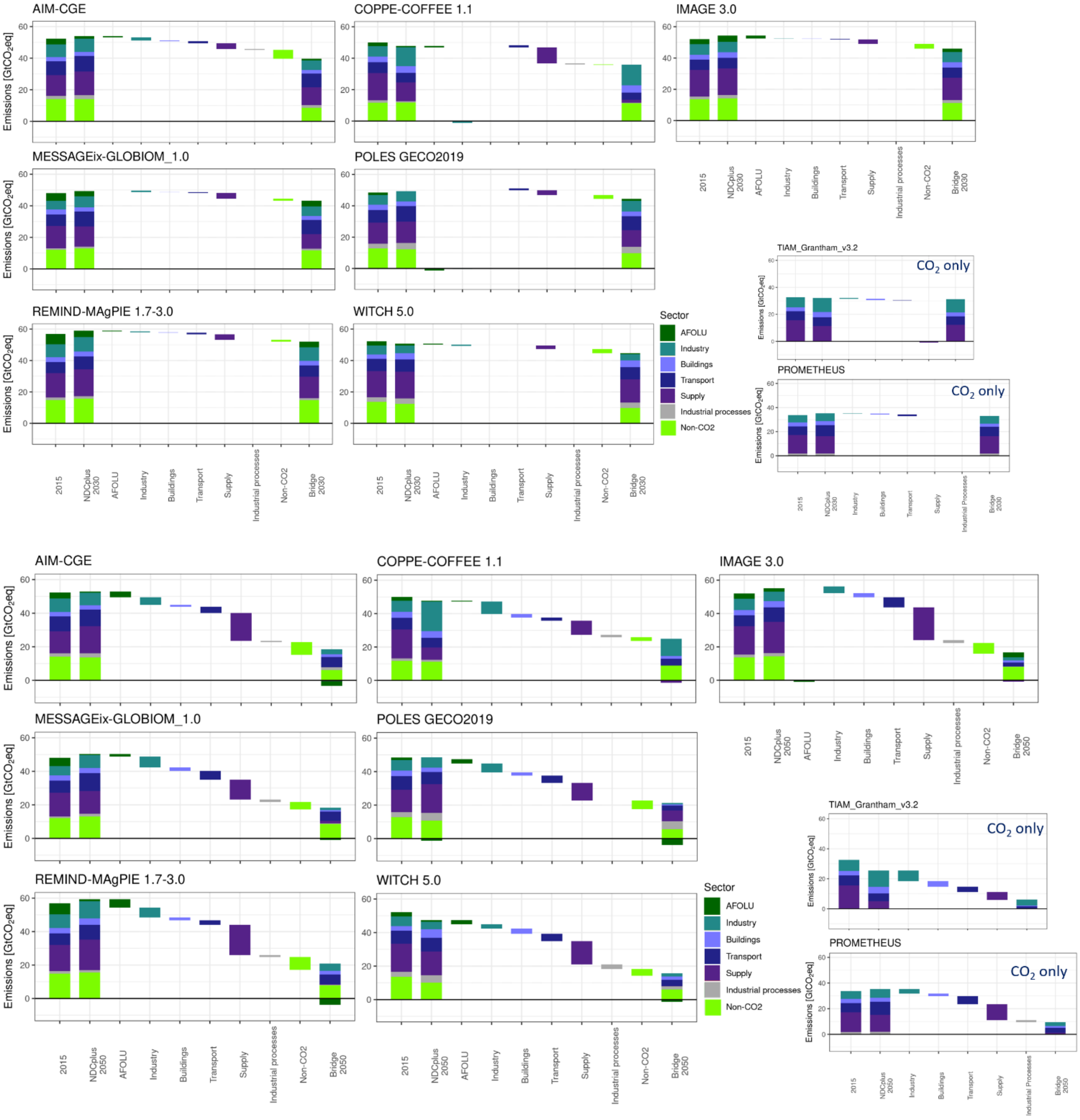


*Supplementary Figure 5: Contribution of each sector to emission reductions between the NDCplus and Bridge scenario* (negative values denote an increase in emissions between NDCplus and Bridge). First bar: Emissions by sector in 2015. Second bar: emissions by sector in 2030 (upper graph) or 2050 (lower graph), under NDCplus. Third - ninth bar: emission reduction in energy supply, industry, buildings, transport, industrial processes, AFOLU, non-$CO_2$ emissions. Last bar: emissions by sector in 2030 (upper graph) or 2050 (lower graph), under Bridge. For TIAM and PROMETHEUS, only $CO_2$ emissions are shown.

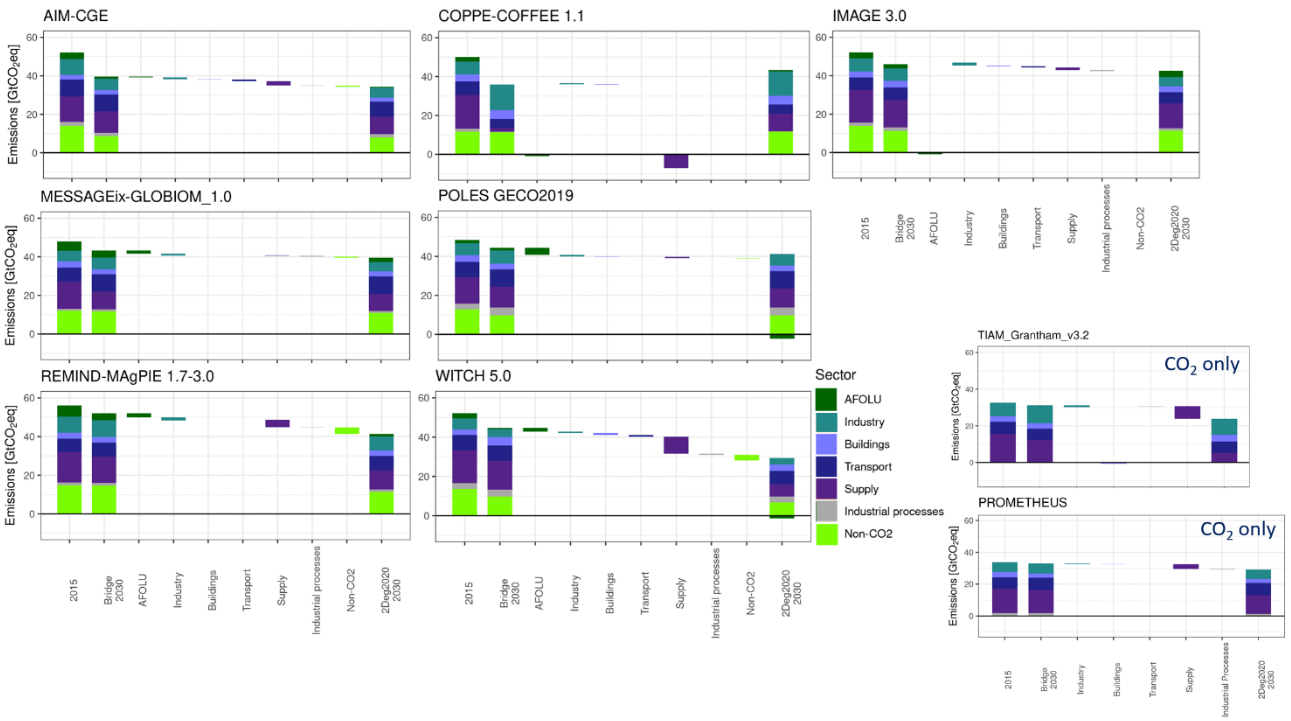


*Supplementary Figure 6: Contribution of each sector to emission reductions between the Bridge and 2Deg2020 scenarios* (negative values denote an increase in emissions between Bridge and 2Deg2020). First bar: Emissions by sector in 2015. Second bar: emissions by sector in 2030, under Bridge. Third - ninth bar: emission reduction in energy supply, industry, buildings, transport, industrial processes, AFOLU, non-$CO_2$ emissions. Last bar: emissions by sector in 2030, under 2Deg2020. For TIAM and PROMETHEUS, only $CO_2$ emissions are shown.

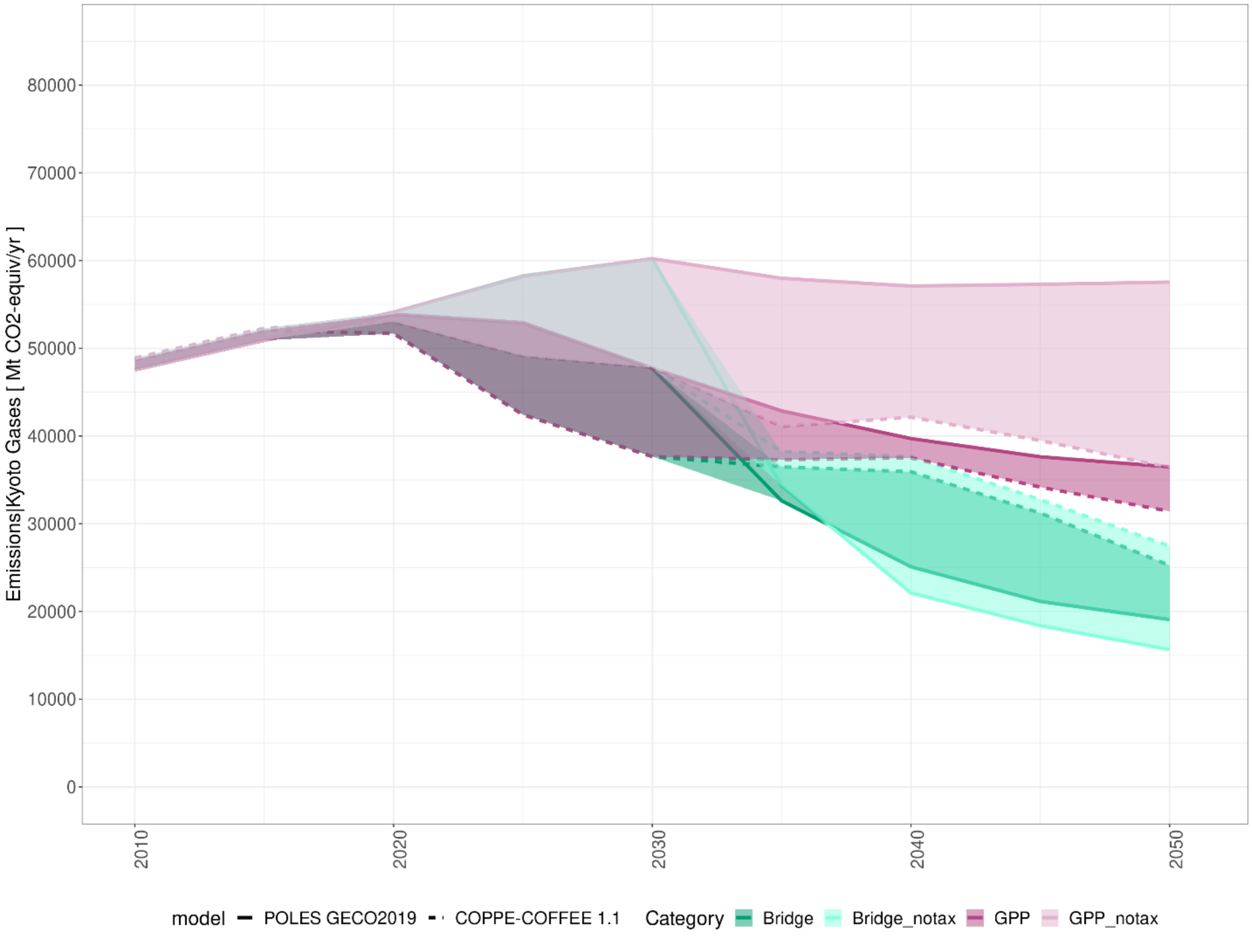


*Supplementary Figure 7: Global greenhouse gas emissions for the sensitivity cases of GPP and Bridge without the carbon tax measure* (measure 7)

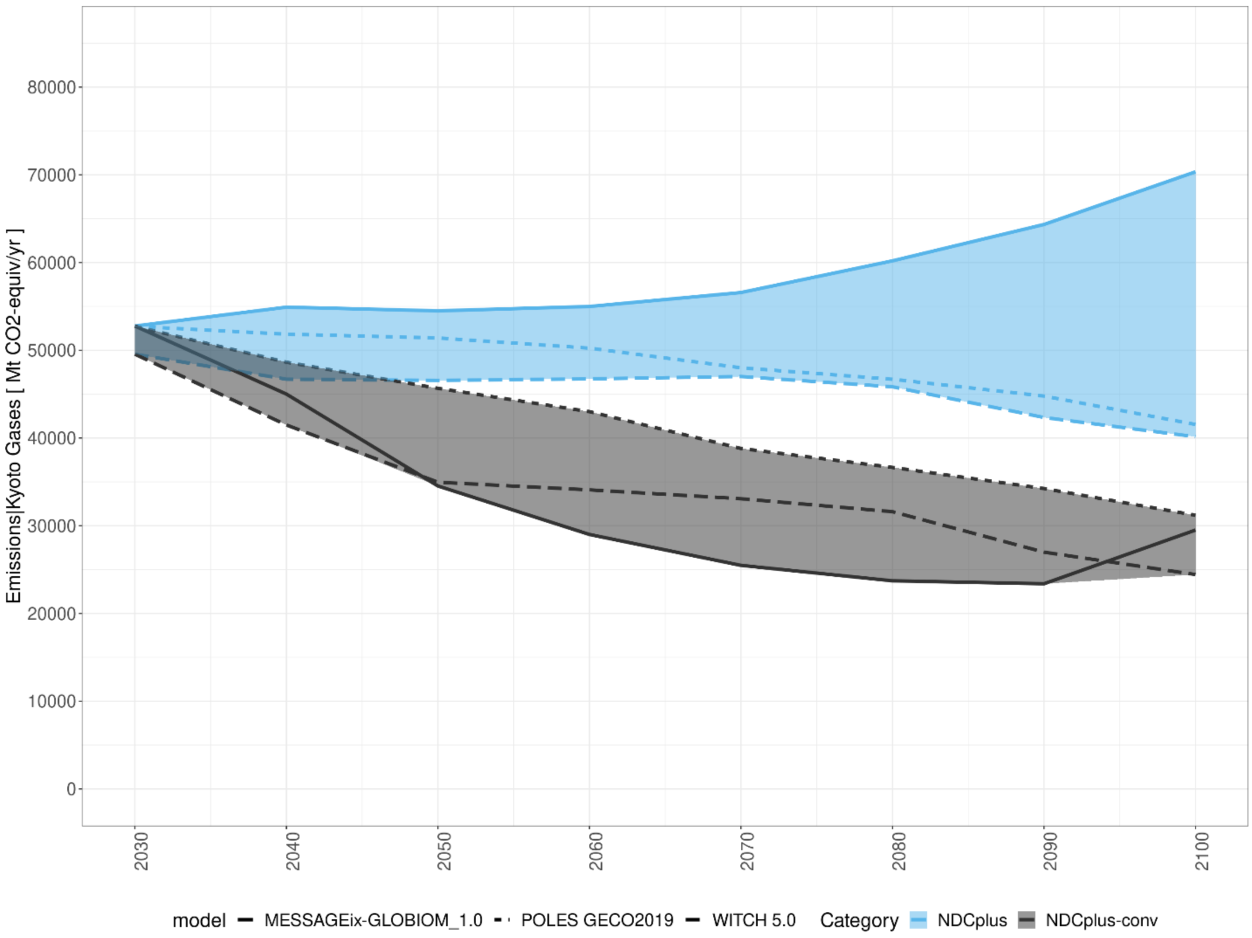


*Supplementary Figure 8: Global greenhouse gas emissions for the NDC variant NDC_2050convergence, as compared to the default NDCplus scenario*

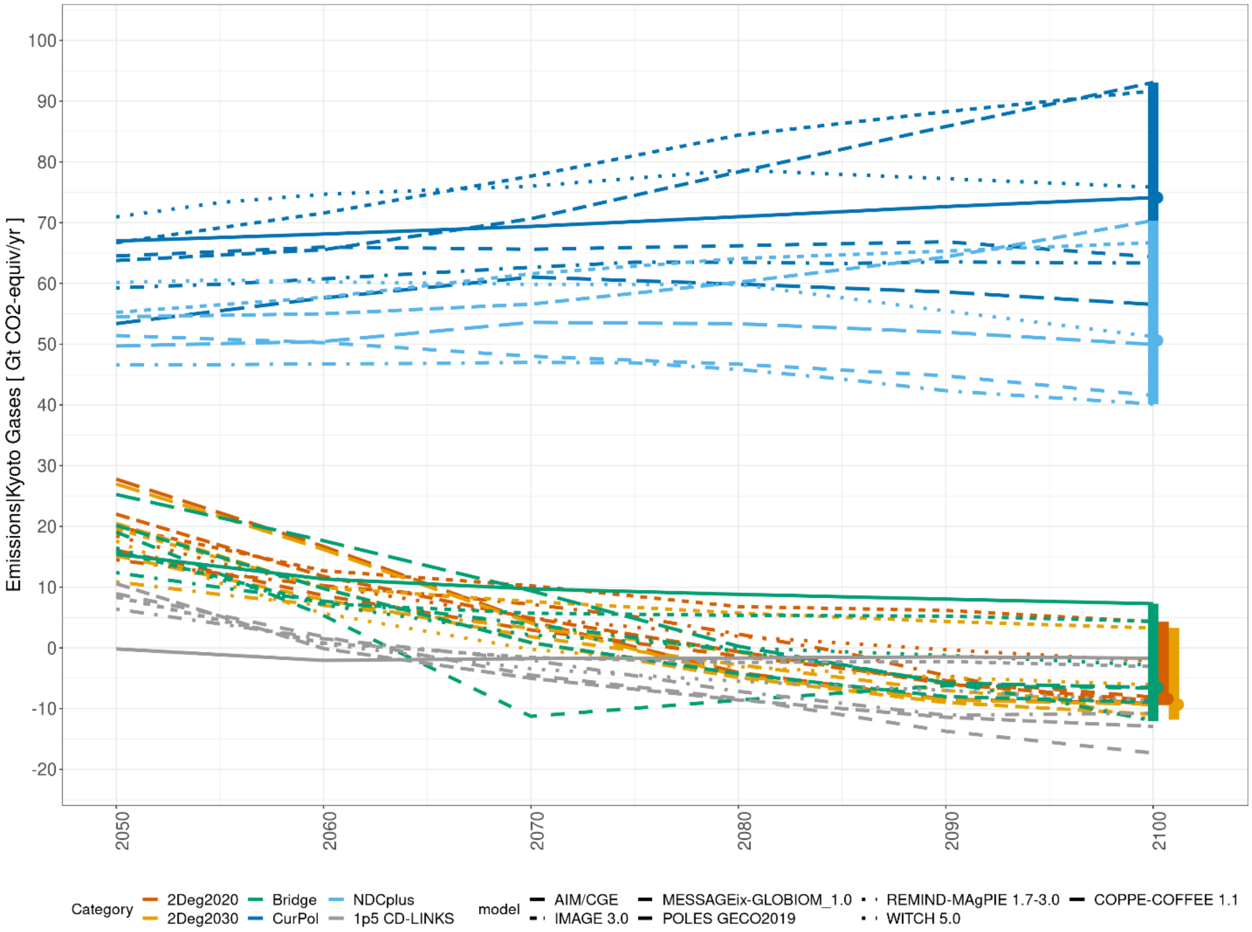


*Supplementary Figure 9: Global greenhouse gas emissions (Gt $CO_2$eq/year) between 2050 and 2100*. *Vertical bars: model range in 2100. Circles: model median in 2100.*

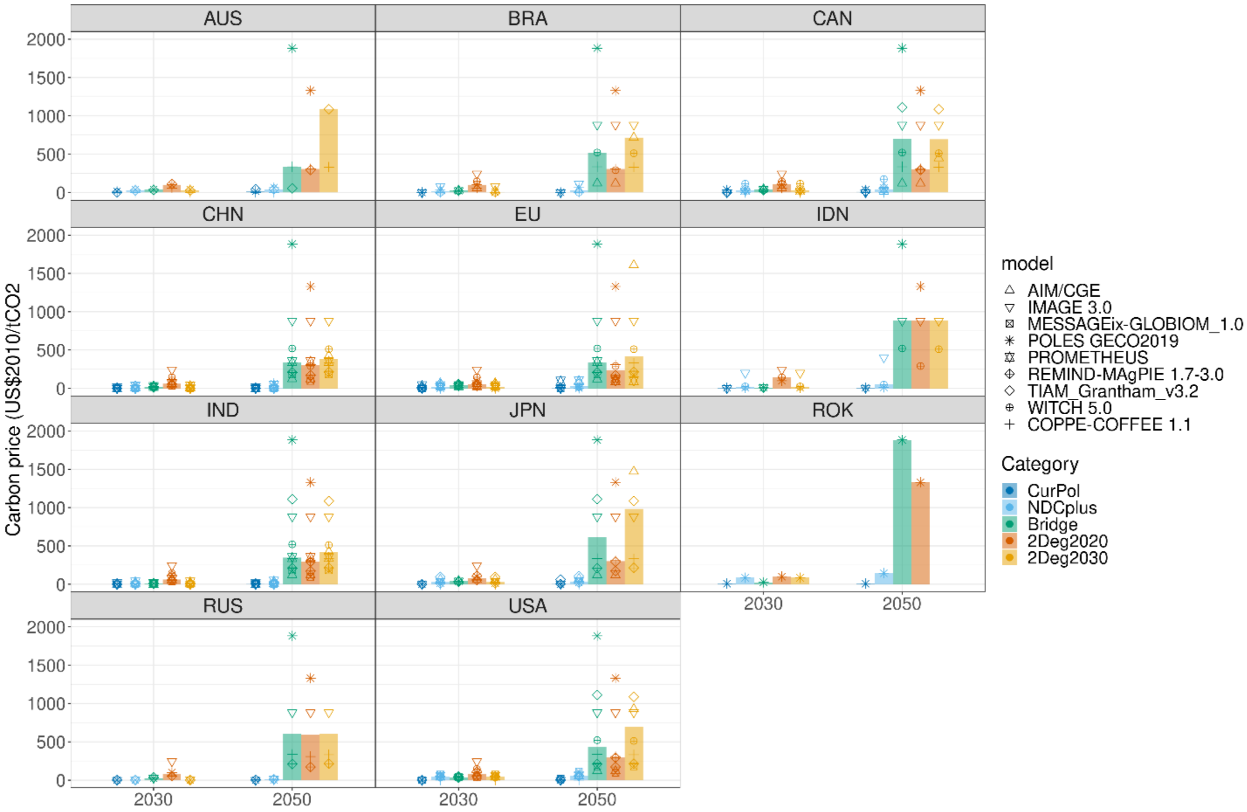


*Supplementary Figure 10: Regional carbon prices (US\$2010/t$CO_2$) in 2030 and 2050, in the CurPol, NDCplus, Bridge, 2Deg2020, and 2Deg2030 scenarios. Bars: model median, symbols: individual models. AUS: Australia, BRA: Brazil, CAN: Canada, CHN: China, EU: European Union, IDN: Indonesia, IND: India, JPN: Japan, ROK: Republic of Korea (South Korea), RUS: Russian Federation, USA: United States of America.*

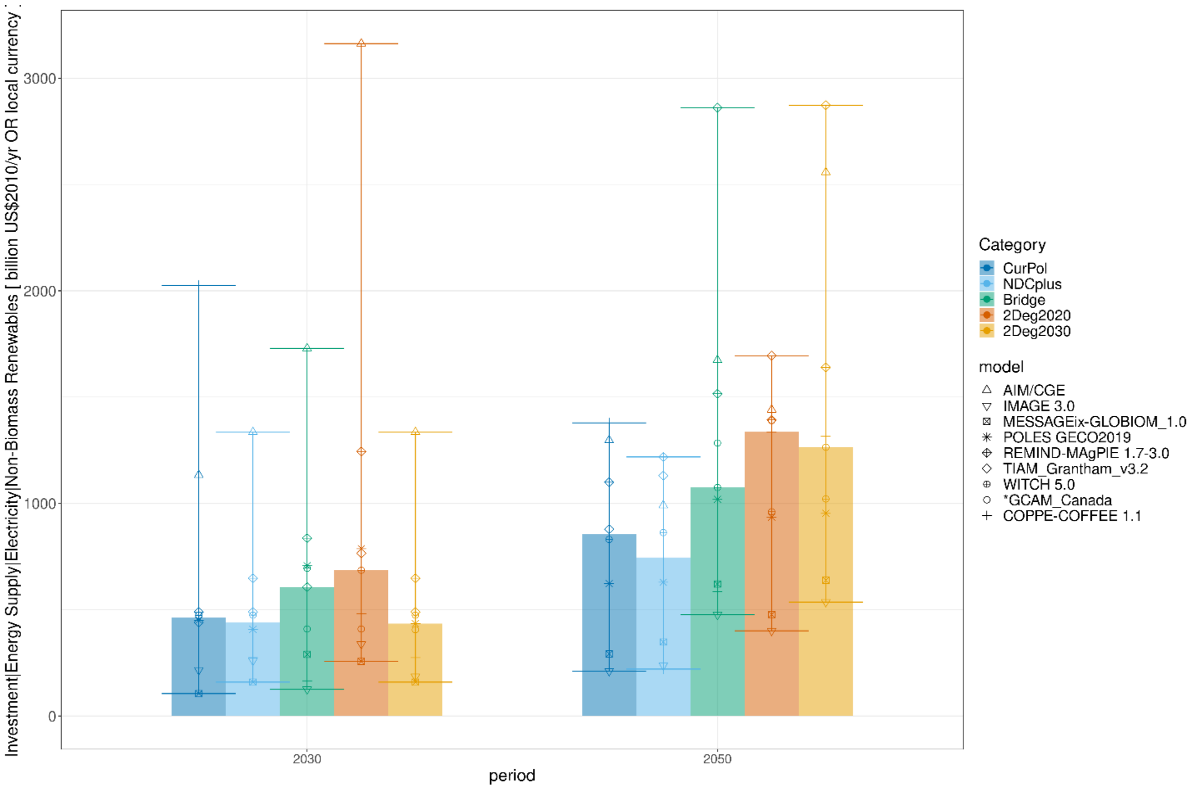


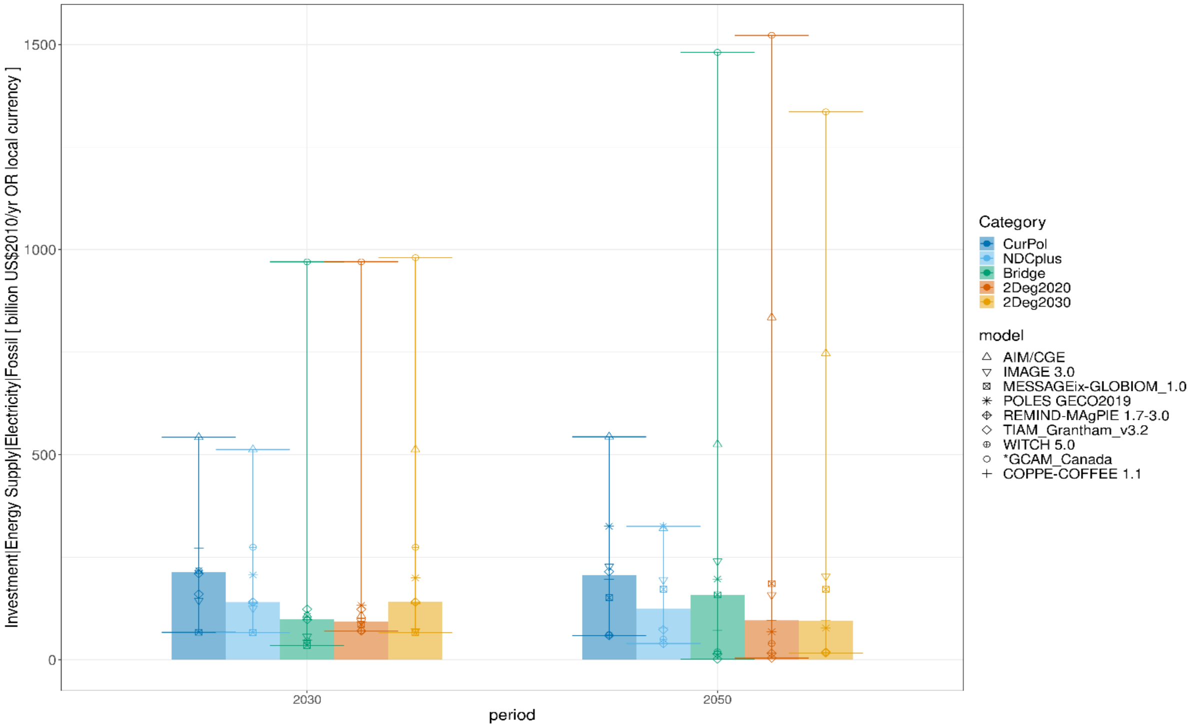


*Supplementary Figure 11: Investments in energy supply – electricity (billion US$2010 per year). Upper graph: non-biomass renewables, lower graph: fossil fuels. Bars show model median, error bars show the full range, and symbols show individual model results.*

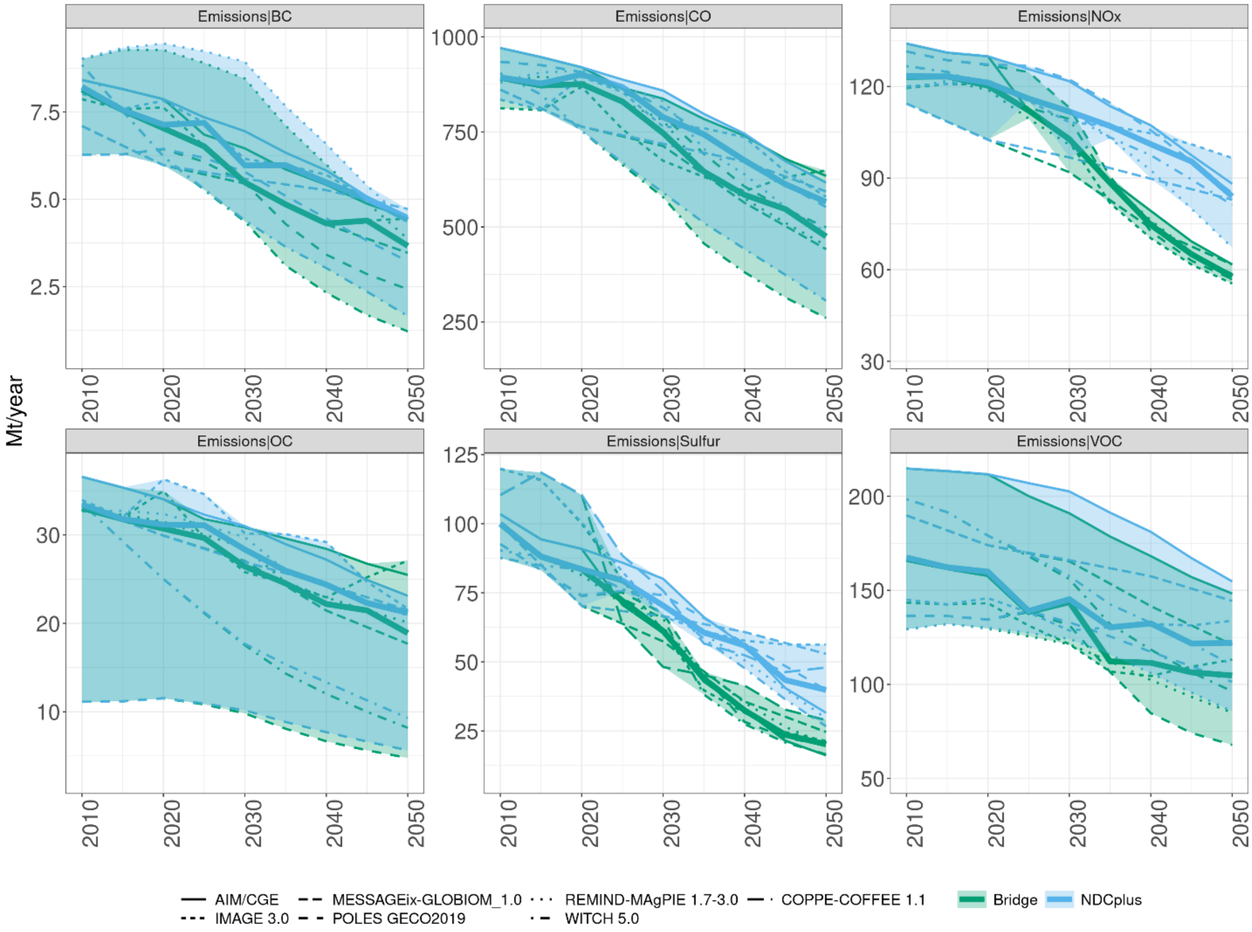


*Supplementary Figure 12: Air pollutant emissions (Mt/year) under the Bridge scenario, compared to NDCplus: black carbon (BC), carbon monoxide (CO), nitrogen oxides (NOx), organic carbon (OC), sulfur, and volatile organic compounds (VOC).*

# Supplementary Methods: COMMIT WP2&3 Scenarios for ratcheting up mitigation ambition

Protocol fourth round – 20 April 2020

## Introduction

In response to the global stocktake under the UNFCCC, scenarios will be developed that represent a ratcheted up mitigation ambition level by Parties to the Paris agreement (hereafter: countries). The scenario suite described here consists of the following scenarios:

- baseline,
- current policy,
- nationally determined contribution (NDC),
- good practice policies,
- bridging, and
- 2 ˚C mitigation scenarios.

The good practice policies and bridging scenarios, which aim to bridge the gap between the ambition levels set out by countries and the required ambition levels to meet the mitigation goals agreed to in the Paris Agreement, are new, and most important. All other scenarios are added for comparison – and could be taken from earlier modelling exercises (although we require consistent model versions).

## Workflow and submission deadlines

Protocol development and scenario submission will take place in two rounds.

In **Round 1**, the national modelling teams got an opportunity to respond to the proposed policies as mentioned in the draft bridging scenario protocol (attached spreadsheet). PBL/PIK/COPPE gathered all comments and used them to construct the final bridging scenario protocol.

In **Round 2**, the final bridging scenario protocol will be distributed to both the national and global modelling teams. This will ensure that a common protocol is followed.

Please use this reporting template.

## Brief description of the scenarios

In line with the global stocktake, the ratcheting up mechanism has been applied in constructing the scenario protocol. This means that the scenarios build upon one another in terms of ambition and modelling assumptions. The Baseline scenario is the least ambitious and the 2 ˚C scenario is the most ambitious.

*Supplementary Table 3. Scenario descriptions*

| Scenario name | Scenario id | Builds upon | Novelty | National model implementation | Global model implementation |
|---|---|---|---|---|---|
| **Baseline** | BAU | None | None | Most likely socio-economic assumptions with no new climate policies after 2010 | SSP2 scenario with no new climate policies after 2010 |
| **Current policy** | CurPol | BAU | Possible update (new information available; old runs can be used) | As BAU scenario, but including implementation of current policies (cut-off date 1 July 2019) | As BAU scenario, but including climate policies based on CD-LINKS/NewClimate/PBL policy database (cut-off date 1 July 2019) |
| **NDC-plus** | NDCplus<br><br>Optional variants:<br>NDC_2050convergence (global models)<br><br>NDCMCS (national models) | CurPol | Update | As CurPol scenario including implementation of NDC until 2030, following the post-2030 extension guidelines thereafter | As CurPol scenario including implementation of NDCs, following the post-2030 extension guidelines thereafter |
| **Good practice policies** | GPP | CurPol | New | As CurPol scenario, including implementation of commonly defined good practice policies until 2050 – taking the value either for developed or for developing countries, and taking the more stringent of the CurPol and GPP values | As CurPol scenario, including implementation of commonly defined good practice policies until 2050 - distinguishing between developed and developing countries, and taking the more stringent of the CurPol and GPP values |
| **Bridging** | Bridge | GPP | New | As GPP scenario including implementation of good practice policies until 2030 to transition to the low carbon budget scenario (2Deg2030) | As GPP scenario including good practice policies until 2030, as identified by national teams, transitioning to the 2 °C scenario (2Deg2030) |
| **2 °C 2020** | 2Deg2020 | CurPol | None | As CurPol with the Implementation of a CD-LINKS carbon budget from 2020 (2020_low) | As CurPol with the implementation of a 2 °C / 2.6 $Wm^{-2}$ climate target from 2020, represented by the CD-LINKS carbon budget: 1000 Gt $CO_2$ over 2011-2100, i.e. NPi2020_1000. |

| Scenario name | Scenario id | Builds upon | Novelty | National model implementation | Global model implementation |
|---|---|---|---|---|---|
| **2 ˚C 2030** | 2Deg2030 | NDCplus | None | As NDCplus with the implementation of a CD-LINKS carbon budget from 2030 (2030_low) | As NDCplus with the implementation of a 2 ˚C / 2.6 $Wm^{-2}$ climate target from 2030, represented by the CD-LINKS carbon budget: 1000 Gt $CO_2$ over 2011-2100, i.e. INDC2030_1000 |

The **Baseline (BAU)** scenario should be a middle of the road socio-economic conditions scenario (preferably SSP2) throughout the century with no additional climate policy.

The **Current policy (CurPol)** scenario assumes the same socio-economic conditions as the BAU scenario. However, it also assumes that climate, energy and land use policies that are currently ratified are implemented (cut-off date 1 July 2019). For global models, this can be based on the updated CD-LINKS protocol. A new version is attached – please refer to the spreadsheet *Input-IAM-protocol_COMMIT_December2019.xlsx*, tab "Protocol CurPol numerical" (note that not all tabs were updated). Note that this update is optional: also the previous current policy scenario (CD-LINKS) could be submitted if needed, if based on the same model version.

The **NDC-plus (NDCplus)** scenario builds further upon the CurPol scenario and assumes that the NDCs are implemented by 2030. After 2030, the scenario should reflect continuation (but not strengthening) of effort (see Post policy period for details). Specifically for China, please incorporate the 'peak' component of the NDC by ensuring that emissions do not increase above 2030 values.

*Optional, additional scenario variants:*

- *NDC_2050convergence - for global models only. In order to explore the implications of a scenario narrative "if the 2050 MCS in all countries become similarly stringent as the NDC targets of OECD countries for 2030", this scenario foresees a global convergence to a globally harmonized carbon price in 2050. See details under 'Post policy period'.*
- *NDCMCS - for national models only. For those countries that have submitted one to the UNFCCC, the MCS target for GHG emissions is implemented by 2050.*

The **Good practice policies (GPP)** scenario builds upon the CurPol scenario and assumes that certain good practice policies as defined in the spreadsheet, which have shown to be effective in some countries, will be implemented globally until **2050. For the list of policies to be implemented, see the spreadsheet *Bridging Scenario GPP list 20 April 2020.xlsx.*** That spreadsheet also contains tabs categorising countries in low / high income or other tiers. A distinction is made between low/medium income (columns K and L of the first tab) and high income countries (columns I and J) in terms of timing and stringency (applied to all model regions). See also the fifth tab, 'Country categorisation', for a classification of all countries with their ISO codes: if the majority of countries in a region is classified as high income, the region can be considered high income (and vice versa for low/medium income). For some measures, we distinguish between three country tiers (columns M and N in the first tab, and see the third tab '7. CarbonPrice' and '16. Afforestation' for the country tiers applying to these measures).

- If CurPol is more stringent than GPP in certain sectors, take that value.

The **Bridging (Bridge)** scenario builds upon the GPP scenario. For the list of policies to be implemented until 2030, see the spreadsheet *Bridging Scenario GPP list 20 April 2020.xlsx.* After 2030, the bridge scenario transitions to the 2 °C scenario (see chapter 5).

The **2˚C (2Deg2020 and 2Deg2030)** scenarios assume that a radiative forcing target of 2.6 $Wm^{-2}$ is reached by 2100 in a cost-effective way. National modelling teams can work with a carbon budget derived from the global carbon budget of 1000 Gt $CO_2$ in the period 2011-2100 (including 2011 emissions), as done in CD-LINKS (the '2020_low' budget for 2Deg 2020 and the '2030_low' budget for 2Deg 2030). Updated national carbon budget numbers for 2015-2050 are attached (*NationalCbudgetsCOMMIT.xlsx)*, including for the teams that did not participate in CD-LINKS, and distinguishing total $CO_2$ and only $CO_2$ from energy and industry for those models that do not represent land use. Global model teams can use the NPi2020_1000 (2Deg2020) and INDC2030_1000 (2Deg2030) scenarios.

# General specifications for all scenarios

## Naming

When uploading results to the IIASA database, scenario names as mentioned in the column Scenario id of Supplementary Table 3 should be used with the extension of the version number, with _V4 for this round. That means: please submit the following scenarios, regardless of whether you have submitted in previous rounds:

- BAU_V4
- CurPol_V4
- NDCplus_V4
    - *NDC_2050convergence_V4*
    - *NDCMCS_V4*
- GPP_V4
- Bridge_V4
- 2Deg2020_V4
- 2Deg2030_V4

## Time horizon

Models are requested to report five year intervals between 2000 and 2020 and 10 year intervals thereafter. Up to 2050 or 2100 based on model specifications. For models that do not have 5-year time steps, targets for e.g. 2035 should be implemented by the nearest (later) year, i.c. 2040. For base year values, use the provided 2015 value if needed.

## Post policy period

For the Current policy, NDC/MCS and Good Practice Policy scenarios, the ambition levels reached in the target year should remain at least constant throughout the rest of the century. This should be implemented by extrapolating the "equivalent" carbon price in 2030, using the GDP growth rate of regions. The equivalent carbon price represents the value of carbon that would yield in a region the same emissions reduction as the NDC policies. For most modelling teams this requires to run a set of (cost-optimal) sensitivity scenarios in order to derive the carbon price that would result in the same reductions as in the NDC cases. Importantly, if a region has a carbon price of zero while implementing the (I)NDC in 2030, please assume a minimum carbon price of 1 $/t$CO_2$ in 2030 (= 8 $/t$CO_2$ in 2100 with 3%/y GDP growth). If a region has a negative carbon price in 2030, offset the trajectory resulting from 1 $/t$CO_2$ to your own 2030 starting point. For land use, a carbon price ceiling of $200/t$CO_2$ should be applied.

*Optional, additional scenario for global models: **NDC_2050convergence***

*In order to explore the implications of a scenario narrative "if the 2050 MCS in all countries become similarly stringent as the NDC targets of OECD countries for 2030", this scenario foresees a global convergence to a globally harmonized carbon price in 2050, at the level of the average carbon prices in OECD countries (which are increasing from 2030 onwards with regional GDP growth rates). Exact calculation of carbon prices: Initially, regional carbon prices after 2030 are taken from the NDC scenario where they are determined by applying the regional GDP growth rate to the effective carbon price in the region in 2030. The "global" carbon price for all years > 2030 is then calculated as the GDP-weighted average carbon price of OECD regions. The regional carbon prices of all regions are then updated to converge from the effective carbon price in the region in 2030 (as in the NDC scenario) to this global level until 2050 (linear increase from regional 2030 carbon price to 2050 global carbon price). They are equal to the global trajectory for all timesteps 2050 and beyond. Only OECD regions with carbon prices higher than the "global" trajectory should stick to their original carbon price trajectory (maximum operator).*

### Regions

Apart from model specific regions, a mapping to the 5 RCP regions should be made by global models.

### Policy coverage in the model

If you are unable to implement the policy or target in the model, please adopt the provided proxy values instead. If that is not possible either, please indicate in the protocol spreadsheet which policies you were not able to capture. Proxy values are provided by IMAGE in the tab 'Indicators'.

For global sectors such as international aviation and shipping, measure 18 is included for aviation (no distinction between countries). For shipping, take the baseline trends (as no current policies are included for that sector).

## Detailed specifications of scenarios

### Updated CD-LINKS scenarios

To a large degree, the scenarios developed in the CD-LINKS project can be used. A mapping between the scenarios can be found in Supplementary Table 4. The third column indicates the novelty of the scenario. The fourth indicates the corresponding CD-LINKS protocol that can be used as reference. The table shows that, with the exception of the good practice policies / bridging and NDC-plus scenario, the protocols from CD-LINKS can be used for all scenarios. Supplementary Figure 13 gives an example of what the increasing ambition emission profiles might look like.

*Supplementary Table 4. Scenario mapping to CD-LINKS protocol*

| Scenario name | Scenario id | Novelty | Protocol (global / national models) |
|---|---|---|---|
| **Baseline** | BAU | Update | CD-LINKS NoPolicy / NoPOL |
| **Current policy** | CurPol | Update | CD-LINKS NPi / NPi |
| **NDC-plus** | NDCplus<br>- *NDC_2050convergence*<br>- *NDCMCS* | Update | New formulation (see this protocol) |
| **Good practice policies** | GPP | New | New (see spreadsheet) |
| **Bridging** | Bridge | New | New (see spreadsheet) |
| **2 ˚C 2020** | 2Deg2020 | Update | CD-LINKS NPi2020_1000 / NPi2020_low |
| **2 ˚C 2030** | 2Deg2030 | Update | CD-LINKS INDC2030i_1000 / INDC2030_low |

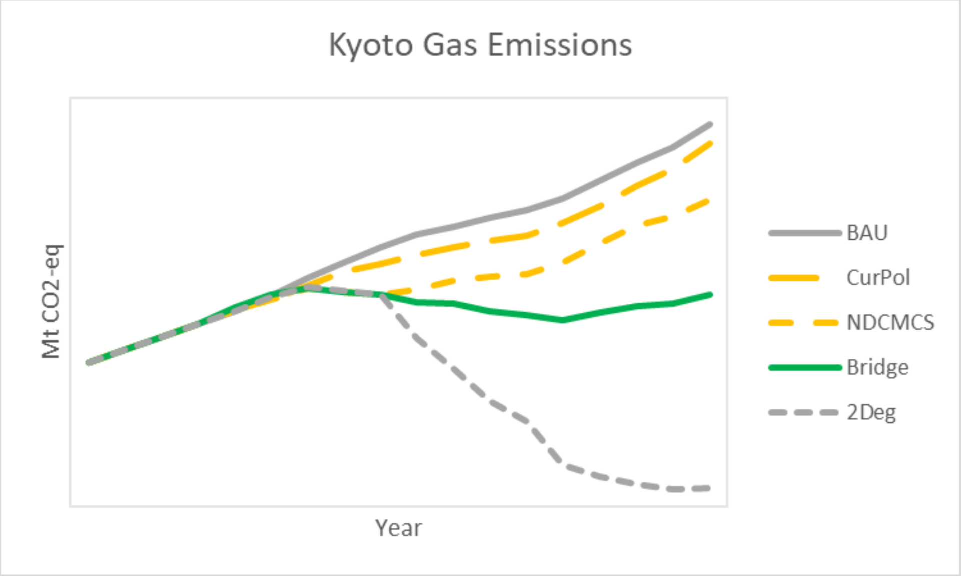


*Supplementary Figure 13: Example of emission profiles (note: the green 'Bridge scenario' actually represents the GPP scenario – the true Bidge scenario would be in between the green line and grey dashed line). The NDCMCS scenario is now optional (for national models) – instead, the NDCplus scenario would be more stringent than shown here.*

## Good practice policies and Bridging scenarios

The list of policies to be implemented is given in the *Bridging Scenario GPP list 20 April 2020.xlsx* spreadsheet (they are based on previous studies as indicated in the sheet "underlying information", which marks as *Kriegler*[1], *Fekete*[2] *or Roelfsema*[3]*)*. Note that these 'policies' are mostly physical measures, without the policy instruments to implement them (given that those are context dependent).

By 9 September, national teams should indicate, for every entry in the spreadsheet, whether they:

1. Believe these policies would be feasible to implement in their country as stated,
2. Are able to implement an adjusted form of the policy (e.g. lower ambition, later implementation year), or
3. Are not able to implement the policy as denoted or an adjusted form of the policy.

Furthermore, teams are encouraged to add optional policies that might apply to their national circumstances. The comments and suggestions have been stored in the tab 'Team comments'.

In the **good practice policies** scenario, the listed policies should be followed until 2030 and in many cases 2050. See 'Post policy period' for assumptions after the last target year.

In the **bridging** scenario, the listed policies should be followed until 2030, after which the scenario should transition smoothly to the 2 °C scenario by remaining within the carbon budget consistent with the 2 °C target (the 1000 $GtCO_2$ for 2011-2100 for global teams / 2030_low for national teams). This should be implemented via a carbon price: please converge from the regionally differentiated 2030 carbon prices listed in tab *7. CarbonPrice* in attached spreadsheet to a global carbon price in 2050 that is in line with the 2 °C carbon budget (in your model). If that implies the targets become infeasible, go for the latest convergence date possible that keeps the target in reach.

**The above implies that the GPP and Bridge scenarios should follow the same pathway until 2030.**

# Supplementary References


1 Kriegler, E. *et al.* Short term policies to keep the door open for Paris climate goals. *Environmental Research Letters* **13**, doi:10.1088/1748-9326/aac4f1 (2018).

2 Fekete, H. *et al.* A review of successful climate change mitigation policies in major emitting economies and the potential of global replication. *Renewable and Sustainable Energy Reviews* **137**, 110602, doi:https://doi.org/10.1016/j.rser.2020.110602 (2021).

3 Roelfsema, M. *et al.* Reducing global GHG emissions by replicating successful sector examples: the 'good practice policies' scenario. *Climate Policy* **18**, 1103-1113, doi:10.1080/14693062.2018.1481356 (2018).
